# New Insights into the Bacterial Fitness-Associated Mechanisms Revealed by the Characterization of Large Plasmids of an Avian Pathogenic *E. coli*

**DOI:** 10.1371/journal.pone.0029481

**Published:** 2012-01-04

**Authors:** Melha Mellata, Jacob T. Maddux, Timothy Nam, Nicholas Thomson, Heidi Hauser, Mark P. Stevens, Suman Mukhopadhyay, Shameema Sarker, Aurélie Crabbé, Cheryl A. Nickerson, Javier Santander, Roy Curtiss

**Affiliations:** 1 The Biodesign Institute, Arizona State University, Tempe, Arizona, United States of America; 2 School of Life Sciences, Arizona State University, Tempe, Arizona, United States of America; 3 The Wellcome Trust Sanger Institute, Hinxton, Cambridge, United Kingdom; 4 The Roslin Institute and Royal (Dick) School of Veterinary Studies, University of Edinburgh, Roslin, United Kingdom; 5 Zoonotic Disease Bacteriology and Mycology Branch Division of Microbiology and Infectious Diseases, National Institute of Allergy and Infectious Disease/National Institues of Health (NIH)/Health and Human Services (HHS), Bethesda, Maryland, United States of America; Institute for Genome Sciences-University of Maryland School of Medicine, United States of America

## Abstract

Extra-intestinal pathogenic *E. coli* (ExPEC), including avian pathogenic *E. coli* (APEC), pose a considerable threat to both human and animal health, with illness causing substantial economic loss. APEC strain χ7122 (O78∶K80∶H9), containing three large plasmids [pChi7122-1 (IncFIB/FIIA-FIC), pChi7122-2 (IncFII), and pChi7122-3 (IncI_2_)]; and a small plasmid pChi7122-4 (ColE2-like), has been used for many years as a model strain to study the molecular mechanisms of ExPEC pathogenicity and zoonotic potential. We previously sequenced and characterized the plasmid pChi7122-1 and determined its importance in systemic APEC infection; however the roles of the other pChi7122 plasmids were still ambiguous. Herein we present the sequence of the remaining pChi7122 plasmids, confirming that pChi7122-2 and pChi7122-3 encode an ABC iron transport system (*eitABCD*) and a putative type IV fimbriae respectively, whereas pChi7122-4 is a cryptic plasmid. New features were also identified, including a gene cluster on pChi7122-2 that is not present in other *E. coli* strains but is found in *Salmonella* serovars and is predicted to encode the sugars catabolic pathways. In vitro evaluation of the APEC χ7122 derivative strains with the three large plasmids, either individually or in combinations, provided new insights into the role of plasmids in biofilm formation, bile and acid tolerance, and the interaction of *E. coli* strains with 3-D cultures of intestinal epithelial cells. In this study, we show that the nature and combinations of plasmids, as well as the background of the host strains, have an effect on these phenomena. Our data reveal new insights into the role of extra-chromosomal sequences in fitness and diversity of ExPEC in their phenotypes.

## Introduction

*Escherichia coli* are versatile bacteria; with the majority being non-pathogenic and considered as commensals. A subset of these bacteria has acquired specific virulence attributes that confer an ability to survive in different niches and cause a broad spectrum of intestinal and extra-intestinal diseases [1], [2]. One of the important aspects of the fitness of *E. coli* is thought to be its ability to survive and persist in a variety of environments, including varied anatomical niches, food, soils, poultry litter, and acidic conditions. Extra-intestinal pathogenic *E. coli* (ExPEC) cause infections outside of their normal intestinal habitat in both mammals and birds, resulting in a considerable economic and public health burden [3], [4]. Major infections associated with ExPEC in humans include urinary tract infections (UTI), newborn meningitis (NBM) and septicemia [4]. In birds, a subgroup of ExPEC, named Avian Pathogenic *E. coli* (APEC), causes a complex of systemic infections, mainly respiratory, often leading to death [4]. The genetic relationship between APEC and other ExPEC of human and animal origin [4] emphasizes the potential zoonotic risk of avian-derived *E. coli* strains. In poultry, isolates associated with fecal matter, environmental contamination and chicken meat products possess virulence gene profiles similar to those causing human outbreaks [5], [6], which suggests that retail chicken may be an important reservoir for *E. coli* causing ExPEC infections in humans.

ExPEC exhibit a high degree of antigenic and genetic diversity, which complicates their diagnosis and the design of cross-protective vaccines [7]. ExPEC are defined by a limited number of O-antigens, with specific O antigens being associated with certain clinical syndromes. For example, *E. coli* from a small number of O serogroups (O4, O6, O14, O22, O75, and O83) cause 75% of urinary tract infections [8] and a limited number of serotypes, principally O1, O2, O78, O8, and O35, are commonly implicated in avian colibacillosis [9], suggesting that not all O polysaccharides have identical virulence properties [10], [11]. The possession of multiple large plasmids is often a defining feature of ExPEC, especially APEC, in which the virulence is partly plasmid-mediated [12], [13], [14], [15], [16], [17], [18], [19].

Although many studies have been dedicated to understanding the pathogenesis of ExPEC, little is known about the mechanisms of their persistence. Since a correlation between the ecology of bacteria and their virulence exists, understanding the mechanisms of fitness and survival of these bacteria in extreme and changing conditions would not only improve our understanding of their persistence, but also will contribute to better design strategies for their prevention and treatments.

Previously, the model APEC strain χ7122 (O78∶K80∶H9), containing three large plasmids pChi7122-1, pChi7122-2, and pChi7122-3, previously named pAPEC-1, pAPEC-2, and pAPEC-3 respectively, and a cryptic plasmid pChi7122-4 (Table 1), has been used to undestand the role of large plasmids in the virulence of ExPEC [12]. Specifically, we determined that both the nature of plasmids and their combinations have an effect on the virulence and the genetic diversity of ExPEC. Although we have clearly determined that pChi7122-1 has a major role in systemic infection of APEC in chickens, the role of the remaining plasmids remained unclear.

**Table 1:** Strains and plasmids used in this study.

Strain/plasmid	Relevant characteristics^a^	Reference
**Strains**		
**χ7122 background**		
χ7122	APEC O78∶K80∶H9, *gyrA* Nal^r^, Str^r^, Sxt^r^	[79]
χ7145	χ7122 (χ289:*hisG-zee*), *rfb* deleted by replacement with *E. coli* K-12 region at 45 min	[10], [80]
χ7167	χ7179 *rfb* ^+^ (O111) prototroph by P1χ2963 lysate χ6206 Strain H30, O26∶H11, SLT-1	[80]
χ7193	χ7179 *rfb* ^+^ (O1), prototroph by P1χ7112 lysate	[10], [80]
χ7367	pChi7122-3, Nal^r^	[12]
χ7368	ΔpChi7122-1, Δ pChi7122-2, ΔpChi7122-3, Nal^r^	[12]
χ7394	pChi7122-1, Nal^r^	[12]
χ7392	pChi7122-2, Nal^r^, Str^r^, Sxt^r^	[12]
χ7561	pAPEC-1, pAPEC-2, Nal^r^, Str^r^, Sxt^r^	[12]
χ7562	pChi7122-1, pChi7122-3, Nal^r^	[12]
χ7274	pChi7122-2, pChi7122-3, Nal^r^, Str^r^, Sxt^r^	[90]
***E. coli*** ** K-12 background**		
χ6092	*E. coli* K-12, Lac^−^ F^−^ Tc^r^	[19]
χ7346	χ6092 pChi7122-1, Tc^r^	[19]
χ7347	χ6092 pChi7122-2, Tc^r^, Str^r^, Sxt^r^	[12]
χ7348	χ6092 pChi7122-3, Tc^r^	[12]
**Plasmids**		
pChi7122-1	103,275 pb plasmid of APEC χ7122	[19], [90]
pChi7122-2	82,676 pb plasmid of APEC χ7122	[19]
pChi7122-3	56,676 pb plasmid of APEC χ7122	[19]
pChi7122-4	4,300 pb plasmid of APEC χ7122	This study

aNal^r^, nalidixic acid resistant; Tc^r^, tetracycline resistant; Str^r^, streptomycin resistant; Sxt^r^, Trimethoprim/sulfamethoxazole resistant.

Since pChi7122-2 and pChi7122-3 do not encode for common ExPEC virulence factors [12], and their roles are considered as minor in systemic infection in chickens [12], we hypothesized that these plasmids could be important in persistence of this bacterial strain in different stressful conditions encountered before and during infections. Therefore, this study aimed to (1) fully sequence and analyze the DNA of plasmids pChi7122-2, pChi7122-3, and pChi7122-4 of APEC strain χ7122; and (2) evaluate the contribution of these plasmids, as well the plasmid pChi7122-1, either individually or in combination, in the bacterial interaction with a model human intestinal epithelial cell line, bile and acid resistance, biofilm formation, and growth in iron-restricted medium and in the presence of different carbon sources. Moreover, since the plasmids can be carried by strains with different backgrounds, we aimed to determine the effect of different host strain backgrounds on plasmid-associated phenotypes. This study presents for the first time the sequence of three plasmids of APEC strain χ7122 and provides new insights into the genetic and phenotypic mechanisms that ExPEC may use for their persistence and survival in stressful conditions.

## Results and Discussion

Genome sequencing has made major contributions to our knowledge of virulence and the evolution of pathogenic bacteria. So far, virulence plasmids associated with ExPEC are ColV, ColBM and Vir plasmids [20]; many of which are already fully sequenced and have been determined as belonging mainly to the IncFIB/FIIA backbone. Although PCR characterization of UPEC and APEC plasmids has revealed the presence of plasmids from other Inc groups [21], studies on their role in ExPEC has been limited. Previously, we examined the role of the three large plasmids of APEC χ7122 in pathogenesis in chickens [12] and sequenced the plasmid pChi7122-1 [19]. We were able to assign roles for pChi7122-1 in the virulence in systemic infection of bacteria; however the roles of pChi7122-2 and pChi7122-3 in APEC χ7122 were equivocal.

### General sequence features of plasmids pChi7122-2, pChi7122-3, and pChi7122-4

In this study, we present the whole DNA sequences of plasmids pChi7122-2 (FR851303), pChi7122-3 (FR851304) and pChi7122-4 (FR851305). The general sequence features of the three plasmids are listed in the Table 2.

**Table 2:** Summary of general characteristics of the three sequenced plasmids of APEC χ7122.

Plasmids	Size (bp)	Inc group	GC%	N° ORFs	Starting codons	Gene function	virulence factors-encoded	ATB
pChi7122-2	82,676	IncFII	52.8	115	ATG (78.26%) GTG (14.78%) TTG (6.08%)	18.26% (known and putative virulence genes) 41.74% (involved in plasmid functions) 4.35% (*IS*s) 19.13% (CHP) 16.52% (HP)	EitABCD	Str^r^, Sxt^r^
pChi7122-3	56,676	IncI2	42.7	86	ATG (86.04%) GTG (6.8%) TTG (5.81%)	26.74% (known and putative virulence genes) 31.39% (involved in plasmid functions) 2.32% (*IS*s) 8.14% (CHP) 31.39% (HP)	Type IV Pil fimbriae	-
pChi7122-4	4,300	ColE2-like	49.3	3	ATG (100%)	66.66% (involved in plasmid functions) 33.34% (CHP)	None	-

ATB, antibiotic; Str^r^, streptomycin resistant; Sxt^r^, Trimethoprim/sulfamethoxazole resistant; -, absent.

**Figure 1:**
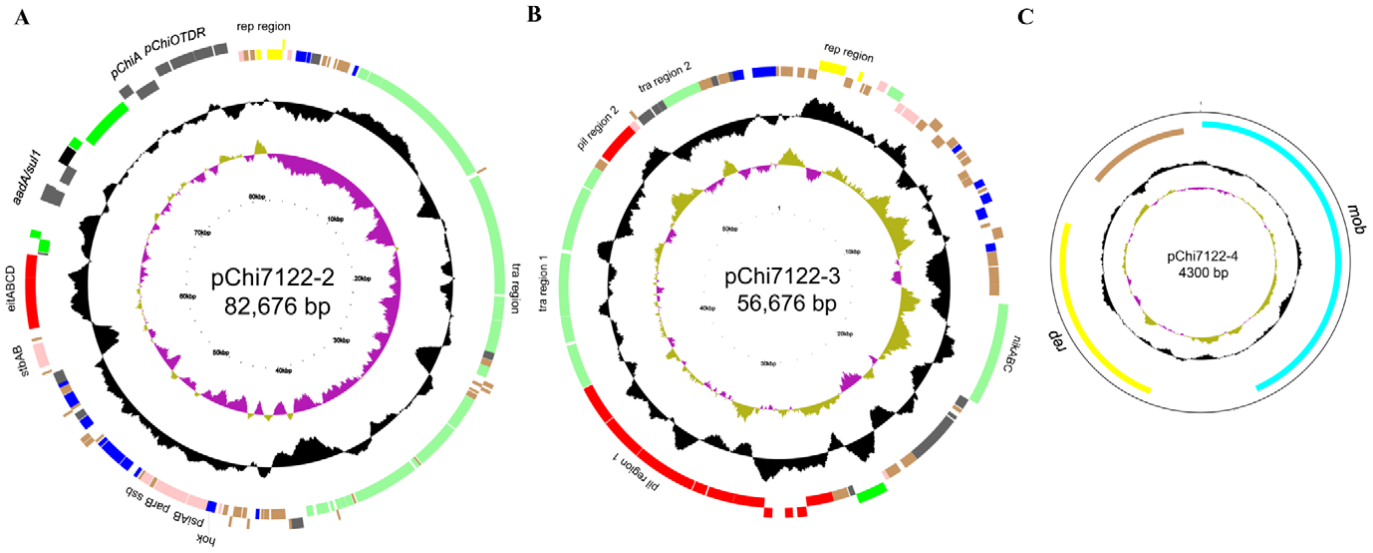
Circular representation of pChi7122-2 (A), pChi7122-3 (B), and pChi7122-4 (C). The different rings represent (from outer to inner) CDS which are color coded by functional group (ring 1 and 2), deviation from average G+C content (ring 3), and GC skew [(G−C)/(G+C); ring 4]. Colors represent the following: red, virulence-associated; green, plasmid transfer; yellow, plasmid replication; grey other functions; brown, hypothetical protein.

Plasmids pChi7122-2, pChi7122-3 and pChi7122-4 consist of 82,676 bp, 56,676 bp and 4,300 bp respectively (Fig. 1, Table 2) and are predicted to encode 115, 86, and 3 coding sequences (CDS) respectively (Table 2, Table S1 and S2); these CDSs include the complete sequences for the iron acquisition system *eitABCD* on pChi7122-2 and type IV fimbriae on pChi7122-3, which have been previously shown to be present on these plasmids by PCR [12]. Analysis of pChi7122-4 revealed 3 CDSs that were predicted to encode plasmid replication and maintenance functions only (Table 2, Fig. 1); consequently we excluded this plasmid from all further experimental analysis.

We assessed the presence of *eitA* gene of pChi7122-2 and two genes of pChi7122-3 (*pilS* and *pilV*) by PCR among 225 pathogenic *E. coli* strains from different origins, including 100 human *E. coli* strains isolated from the main clinical extra-intestinal sources (50 UTI and 50 non-UTI), 80 APEC, and 45 human enteric pathogenic *E. coli*. PCR results show that *eitA* was present in 10% of non-UTI human isolates and 5% of APEC strains, but was absent in other groups. The genes *pilS* and *pilV* of pChi7122-3 were detected in 8.75% of the APEC group and in 10% of human UTI isolates, respectively. The low prevalence of pChi7122-2 (*eitA*) and pChi7122-3 (*pilS* and *pilV*) genes among other ExPEC of human and avian origin, as determined by PCR, could indicate the recent acquisition of these genes by these *E. coli* strains, enabling them to inhabit new niches.

Our past work has determined that plasmids pChi7122-2 and pChi7122-3 are self-conjugative [12]. Herein, their sequences analysis has revealed the presence of genes required for their transfer (Fig. 1, Table S1 and S2). The transfer region of pChi7122-2 is about 34 kb consisting of 24 *tra* and 9 *trb* genes (Table S1) and is identical to the one of the virulence plasmid pAA (FN554767.1; 99% identity with 92% coverage), whereas the *tra* region of pChi7122-3 has the same organization as its equivalent in *E. coli* conjugative plasmid IncI2 R721 (AP002527.1); it contains 11 *tra/trb* genes, grouped in two clusters separated by two *pil* genes (Fig. 1, Table S2). pChi7122-3 also harbors genes *nikB*, *nikC*, and *nikA* for relaxome formation involved in plasmid transfer [22], [23] (Fig. 1, Table S2).

Among the three plasmids, only pChi7122-2 carries antibiotic resistance genes (MM2-101, MM2-102 and MM2-103) (Table S1). These genes encode for a dihydropteroate synthase (*sul1*) [24], a GCN5-related N-acetyl transferase [25] and a streptomycin 3′-adenylytransferase (SP-R) (*aadA*) [26], respectively. The phenotypic expression of streptomycin and sulfonamide (trimethoprim/sulfamethoxazole) resistance in strains containing pChi7122-2 has been determined by disk diffusion tests. Although streptomycin has only limited current usage in clinical medicine, it remains important for therapy of, and growth promotion in, animals and bacterial disease control in plants [27]. It was suggested that sulphonamide resistance genes can be transferred from commensal bacteria via integrons, transposons or plasmids, into more virulent bacteria in the intestine [28].

Comparative analysis of the pChi7122 plasmids with those in the public databases using BLASTn showed that pChi7122-2 shares high homology with plasmids from *Shigella sonnei* (pEG356), an urinary *E. coli* isolate (pHK01), EAEC (pAA), and *K. pneumoniae* (pKF3-70), respectively (with 100% identity and 70% coverage). The pChi7122-3 genome has shown homology with only one plasmid, the *E. coli* plasmid R721 (with 99% identity and 90% coverage), which includes the type IV fimbriae *pil* operon [29], [30] and shufflon [31], [32]. Mauve alignment of pChi7122-2 and pChi7122-3 with their respective homologous plasmids confirmed these homologies (Fig. S1).

**Figure 2:**
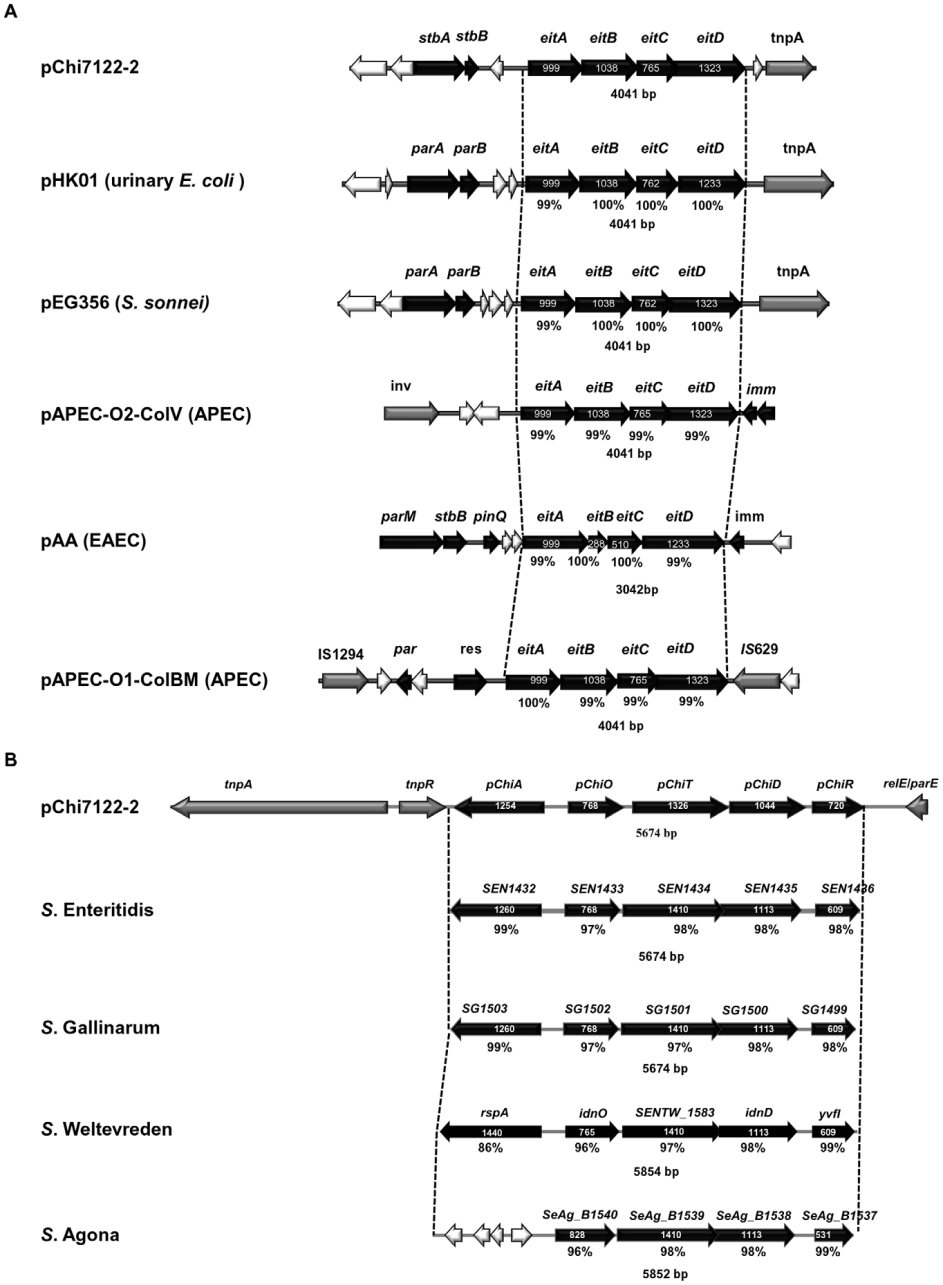
Comparison of physical and genetic maps for *eitABCD* and sugar pathway regions. The *eitABCD* region from pChi7122-2 was compared to its equivalent in pHK01 (HM355591.1), pEG356 (FN594520.1), pAPEC-O2-ColV (NC_007675.1), pAA (FN554767.1), and pAPEC-O1-ColBM (NC_009837.1) (**A**); and sugars pathways genome region *pChiA pChiOTDR* in pChi7122-2 to its equivalent found in the genomes of *S*. Enteritidis (AM933172.1), *S*. Gallinarum (AM933173.1), *S.* Weltevreden (FR775220.1) and *S*. Agona (CP001138.1) respectively (**B**).

The plasmid pChi7122-2 and its homologous plasmids have a 4 kb region in common, which encodes for the ABC iron uptake locus *eitABCD* (Fig. 2A), previously described in two other APEC plasmids, pAPEC-O2-ColV [17] and pAPEC-O1-ColBM [15]. A DNA comparison of the regions of *eitABCD* of the six plasmids has shown that with the exception of pAPEC-O2-ColV, this region is located downstream of the *par* region of plasmids and is flanked by the transposon *tnpA* gene in pChi7122-2, pHK01, and pEG356 respectively and by an insertion sequence *IS*629 in pAPEC-O1-ColBM (Fig. 2A) which could explain the dissemination of *eitABCD* among genomes of these bacteria. We were unable to detect the iron-uptake phenotype expression of *eitABCD* genes using CAS agar medium [12], even though it was efficient in revealing those of pChi7122-1 and chromosomally-encoded systems. Therefore in this study, we extended the analysis by testing the growth of strains, with and without the three plasmids, in iron-limited medium alone or supplemented with either FeSO_4_, heme or hemoglobin. Our results show that only pChi7122-1 increased the growth of strains in iron-sequestered environments (Fig. S2). The ability to acquire iron from heme and hemoglobin could be related to the autotransporter Tsh encoded by pChi7122-1 [33], which has previously been reported to bind to red blood cells [34]. Future studies are needed to determine conditions of expression of *eitABCD*, such as under in vivo conditions.

### New putative sugar utilization pathways identified in pChi7122-2

An important aspect of pathogenesis is the ability of bacteria to adapt their metabolism to the available nutrients by coordinating their metabolism with their life cycle [35]. Recent reports have shown that in the intestine, both commensal and enterohemorrhagic *E. coli* (EHEC) require multiple carbon metabolic pathways [36], [37].

**Figure 3:**
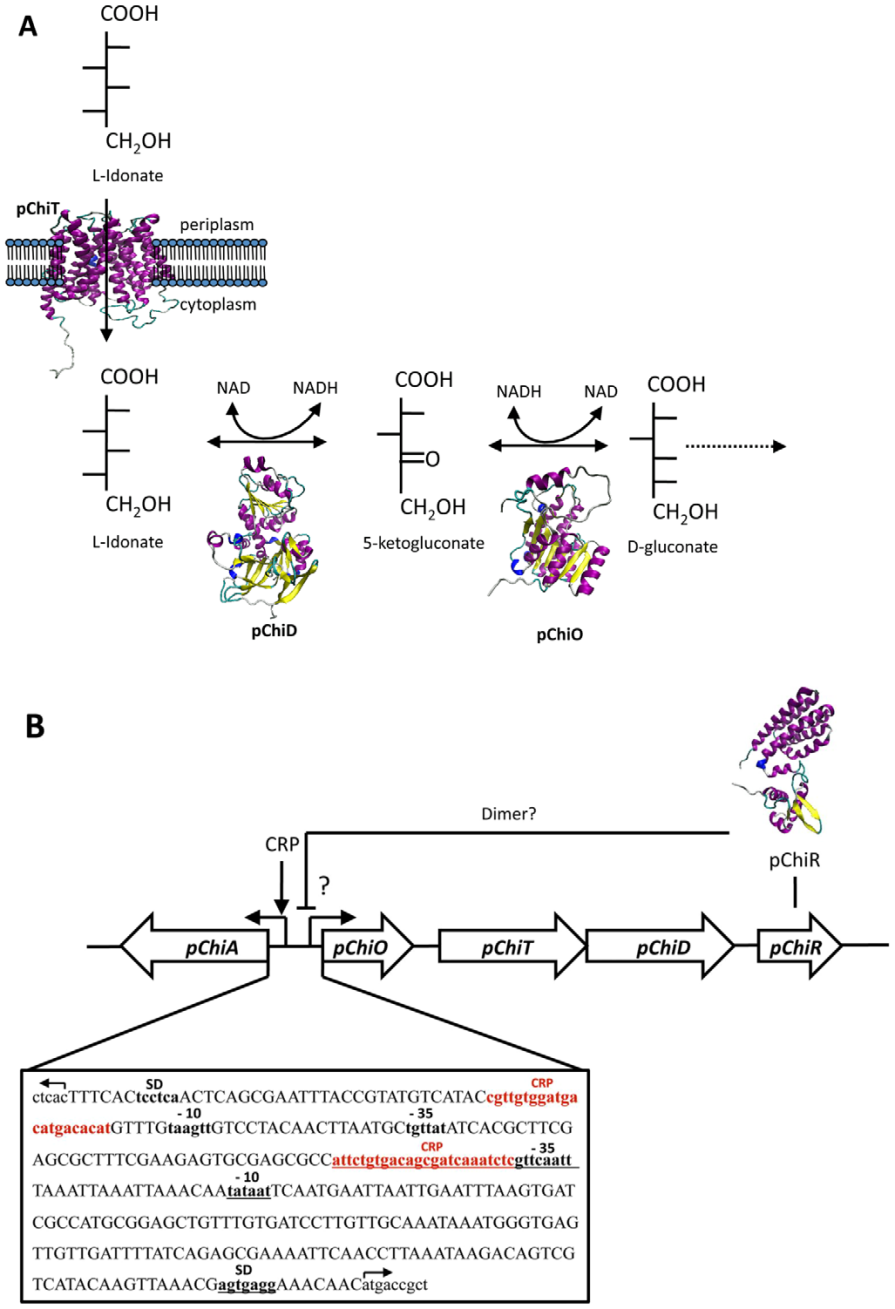
Plasmid pChi7122-2 putative sugar utilization pathway. Illustration of the proposed biochemical pathway for putative sugar utilization encoded by the operon *pChiOTDR* (**A**), the predicted 3D model and function of each enzyme are presented; and diagrammatic representation of the genetic organization of the putative sugar utilization operon *pChiA pChiOTDR* (**B**). The two promoter regions identified are indicated with arrows. Bioinformatic analysis indicated that pChiR may act as a transcriptional regulator of *pChiOTDR* genes. The promoter region contains independent CRP binding boxes indicated in red. The promoter elements for *pChiOTDR* (P_pchi_) located in the positive DNA strand are underlined. The promoter elements for *pChiA* (P_pChiA_) in the negative strand are in bold and red without underlining.

In this study, DNA sequence analysis of pChi7122-2 has revealed the presence of two systems of sugar utilization pathways. This system, with two divergent operons, consists of a gene for a starvation-sensing protein (*pChiA*) located in the opposite orientation to four successive genes *pChiOTDR* (Fig. 2B, 3). These genes have no significant homology with DNA sequences of other *E. coli* available on public databases, as determined by MegaBLASTn search analysis, but share 94% homology (with 100% coverage) with the chromosomal DNA sequence of genomes of *Salmonella* Enteritidis (AM933172.1), Gallinarum (AM933173.1), Weltevreden (FR775220.1) and Agona (CP001138.1), respectively (Fig. 2B). The sequence analysis of this region in these *Salmonella* serovars has determined that, with the exception of *S*. Agona, in which the *pChiA*-equivalent gene is truncated, the organization of the *pChiOTDR* homologous genes in the genome of the four pathogens is the same (Fig. 2B). The identities of the proteins translated by these genes were between 86%–99% (Fig. 2B, Table S3, and Fig. S3).

The putative functions and the predicted 3-D structures of the *pChiA* and *pChiOTDR* gene products, determined by Blast-PSI and HHpred [38], show that *pChiA* encodes for a bifunctional dehydratase that utilizes both D-mannonate and D-altronate as substrates [39] and *pChiOTDR* encode for a gluconate 5-dehydrogenase, pChiO; an exonate sugar transport, pChiT; an L-idonate 5 dehydrogenase, pChiD; and a regulator protein GntR-like, pChiR, respectively (Table S3, Fig. 3A). Two promoter regions, P_pChiA_ and P_pChi_, with independent cAMP receptor protein (CRP) binding boxes [40], [41], were detected in the promoter region of *pChiA* and *pChiO* (Fig. 3B). Bioinformatic analysis indicated that pChiR is a putative transcriptional regulator from GntR family [42]. In the absence of glucose, the preferred carbon source for *E. coli*, the CRP would activate the pChi7122-2 sugars pathways [40], [41]; whereas pChiR would have an opposite effect. It is known that colonic mucus contains several sugar acids that represent an important source of nutrients and that genes involved in the catabolism of N-acetylglucosamine, sialic acid, glucosamine, gluconate, arabinose, and fucose are expressed in both commensal *E. coli* and EHEC [36]. It has also been reported that UPEC bacteria grown in urine express enzymes for catabolism of sialic acid, gluconate, xylose, and arabinose [43] and genes involved in the transport of gluconate and related hexonates are up-regulated in *S.* Typhimurium in macrophages [44], suggesting that the new pChi7122-2 sugar pathways could also be important either in the pathogenesis of APEC, as well as in *Salmonella* serovars Enteritidis, Gallinarum, Weltevreden and Agona or in their persistence in different hosts.

Compared to the chromosomal *E. coli* K-12 L-idonic acid pathway encoded by the *gnTII* genes, *idnK idnDOTR* [45], the genes of the operon *pChiOTDR* of pChi7122-2 have no significant homologies at the DNA level and share some sequence identity at the protein level (Table 3); moreover, the position of the gene of L-idonate 5 dehydrogenase is different in the two distinct gene clusters. Intriguingly, the gluconate kinase gene, *idnK*, of GnTII pathway [45] is absent in the pChi7122-2 pathway and is substituted by the gene of the starvation sensing protein, *rspA-like* [46] *pChiA* which is essential for survival of bacteria in limited nutrient conditions. The gene encoding the regulatory protein GntR in the GnTII pathways, exhibits no significant homology at both DNA and protein levels with its counterpart in pChi7122-2 (*pChiR*) (Table 3). In this study, although we have shown that strains have better growth in media with glucoronic acid than with other sugars tested (Fig. S4), there were no significant differences between strains with and without the plasmid pChi7122-2. The functionality of the sugar utilization pathway genes located on pChi7122-2 would be more apparent in *gntII*-operon-deleted strains [45], or by evaluation of their expression under in vivo conditions, such as using the selective capture of transcribed sequences (SCOTS) method [47]. Future studies will be conducted to determine the conditions of their expression and their eventual role in both APEC and *Salmonella* serovars.

**Table 3:** Comparison of the pChi7122-2-encoded sugar pathway operon with GntII L-idonic pathway of *E. coli K-12*.

L-idonic acid-like catabolism pathway of pChi7122-2 *rspA pChiOTDR*			L-idonic acid catabolism pathway GntII of *E. coli* K-12 *idnK idnDOTR*			
GI-Numbers	Gene symbols	Gene product	DNA homology	% AA identity/%positive	Expect	Gene (Accession no.)
MM2_107	*pChiA*	Starvation sensing protein	NSH	-	-	
MM2_108	*pChiO*	Gluconate 5-dehydrogenase	NSH	48%/64%	1e-70	*idnO* (AAC77203.1)
MM2_109	*pChiT*	The major facilitator superfamily protein	NSH	23%/46%	0.033	*idnT* (AA77222.1)
MM2_110	*pChiD*	L-idonate 5-dehydrogenase	NSH	46%/69%	1e-97	*idnD* (NP_418688.1)
MM2_111	*pChiR*	Regulatory protein GntR	NSH	None	None	*idnR* (NP_418685.1)

AA, amino acid; -, absent; NSH, no significant homology.

### Diversity of plasmids-associated fitness phenotypes and the effect of host strain background on their expression

The genomic diversity among ExPEC isolates has been described and multiple factors have been linked to their virulence [48], [49]. However, a systematic analysis of ExPEC phenotypic diversity has not been done previously. In this study, the large plasmids-associated phenotypes related to fitness of ExPEC bacteria as well the effect of host strain backgrounds were investigated.

Intestines are suspected to be a primary reservoir of ExPEC strains causing diseases in both humans [50] and chickens [5]. To determine if large plasmids would increase the fitness of their carriers in the gastrointestinal (GI) tract environment, we assessed the ability of strains to colonize intestine cells and resist both acid and bile, attributes that allow enteric bacteria to live and persist in the intestine of their host [51].

#### APEC strain χ7122 associates with and invades into intestinal epithelial cells without affecting the distribution of the tight junction protein ZO-1

**Figure 4:**
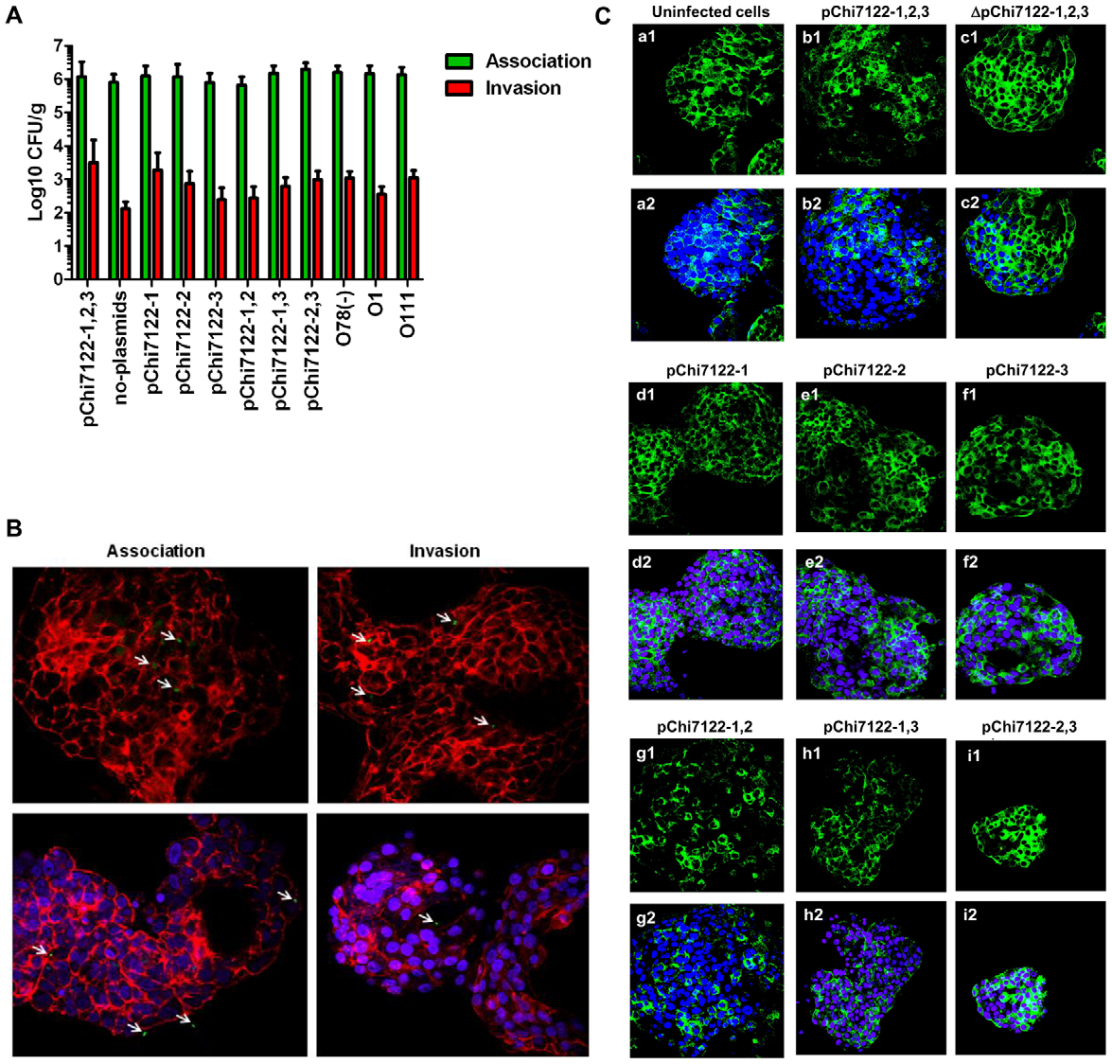
Comparative of association and invasion of strains with 3-D INT-407 cells. Mean of Log CFU/ml and standard deviation of bacteria association and invasion with cells (**A**) Representative confocal laser scanning micrographs showing association, and invasion of 3-D INT-407 cells with χ7122 and derivatives (**B**). Bacteria are marked in green, while the blue and red colors represent cell nuclei labeled with DAPI and F-actin cytoskeleton labeled with phalloidin, respectively; and ZO-1 staining of non-infected and infected 3-D INT-407 with plasmid-derivative strains (**C**). Non-infected 3-D INT-407 aggregates (**a**) or 3-D INT-407 aggregates infected for 2 h with wild type (χ7122) (**b**), ΔpChi7122-1,2,3 (χ7368) (**c**), pChi7122-1 (χ7394) (**d**), pChi7122-2 (χ7392) (**e**), pChi7122-3 (χ7367) (**f**), pChi7122-1,2 (χ7561) (**g**), pChi7122-1,3 (χ7562) (**h**), and pChi7122-2,3 (χ7274) (**I**). The ZO-1 antigen is marked in green, while the blue color represents cell nuclei labeled with DAPI. Images are presented with (indicated as “2”) and without (indicated as “1”) DAPI labeling for clarity purposes. Images are based on 400× magnifications. Arrows indicate the bacteria stained in green; Abbreviations used are: pChi7122-1,2,3 = pChi7122-1, pChi7122-2, and pChi7122-3; pChi7122-1,2 = pChi7122-1 and pChi7122-2; pChi7122-1,3 = pChi7122-1 and pChi7122-3; pChi7122-2-3 = pChi7122-2 and pChi7122-3.

Some APEC strains are genetically similar to human ExPEC, especially to uropathogenic *E. coli* (UPEC) [52], and could cause human diseases [53]. Herein, we investigated the ability of APEC-derivative strains to associate with, and invade into, human cells of the kind that may be targeted by human ExPEC bacteria during their commensal life cycle in the intestine. The intestine is suspected to be a reservoir of ExPEC that cause infections in humans [50]. Since APEC strains are now considered as potential food-borne pathogens that could be transmitted to humans via poultry products [4], [6], [28], we aimed to investigate the interaction of APEC-derivative strains with 3-D organotypic models of human intestinal epithelial cells. The 3-D model of intestinal epithelium used in this study has been shown previously to mimic the in vivo parental tissue more closely than monolayer cultures with regard to morphology and function [54]. The highly differentiated character of the 3-D intestinal cells is reflected in the presence of distinct apical and basolateral polarity, increased expression and better organization of tight junctions, extracellular matrix, and brush border proteins, highly localized expression of mucins, and multiple epithelial cell types relevant to those found in vivo [55]. Our data showed that APEC-derivative strains were able to associate with, and invade into, human intestinal epithelial cells, and large plasmids did not have significant effect on these characteristics (Fig. 4). Although tight junctions efficiently restrict most microbes from penetrating into deeper tissues and contain the microbiota, some pathogens have developed specific strategies to alter or disrupt these structures as part of their pathogenesis, resulting in either pathogen penetration, or other consequences such as diarrhea. In this study, evaluation of different APEC-derivative strains for their interaction with 3-D human intestinal epithelial cells, showed that although these strains attached and invaded into these cells, they did not disturb their tight junctions, based on immunofluorescence evaluation (Fig. 4). These data suggest that invasion of the intestine and dissemination would not occur through intercellular transportation of the bacteria, which could potentially disseminate through transcellular transportation, a mechanism used by meningitis-causing bacteria, including *E. coli* K1 to invade brain microvascular endothelial cells (BMECs) [56]. These bacteria could live as commensals in the intestines from where they shed and cause diseases in different hosts or other sites of the same host.

#### Role of plasmids in bile and acid resistance

**Figure 5:**
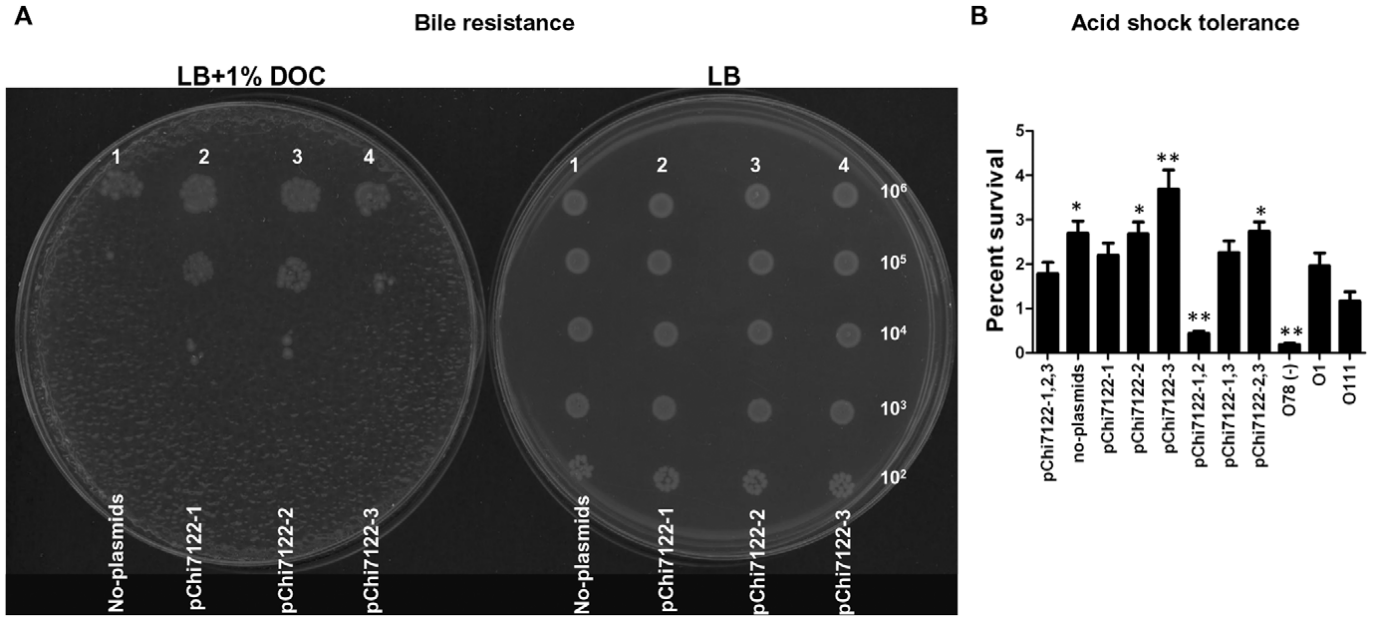
Bile and acid tolerance of strains. Bile sensitivity assay for *E. coli* K-12 and derivatives, no-plasmids (χ6092), pChi7122-1 (χ7346), pChi7122-2 (χ7347), and pChi7122-3 (χ7348). Five-microliters of serial ten-fold dilutions of each strain were spotted on both LB and LB +1% (w/v) DOC agar plates. The approximate numbers of bacteria present in each dilution are indicated on the right side of the plate (**A**). Percent acid survival of wild-type derivative strains in acid shock for 18 hours (**B**). Abbreviations used are: pChi7122-1,2,3 = pChi7122-1, pChi7122-2, and pChi7122-3; pChi7122-1,2 = pChi7122-1 and pChi7122-2; pChi7122-1,3 = pChi7122-1 and pChi7122-3; pChi7122-2-3 = pChi7122-2 and pChi7122-3.

Mechanisms associated with bile resistance in bacteria are LPS synthesis, expression of efflux pump genes and regulatory genes such as *marAB* and *phoPQ* [51]. In this study, we have shown that all wild-type derived strains tested were resistant to deoxycholate (DOC), one of the most abundant bile salts in humans (data not shown); whereas the group of strains derived from *E. coli* K-12 behaved differently (Fig. 5A). Although, *E. coli* K-12 was sensitive to the bile, its plasmid derivative strains χ7346 (pChi7122-1) and χ7347 (pChi7122-2) had increased survival in LB agar media with 1% (w/v) DOC as compared to their parent χ6092. The strain χ7348 (pChi7122-3) was as sensitive to bile as its parent strain χ6092 (Fig. 5A). According to our results APEC χ7122 strain better tolerates the presence of bile salts in the media then *E. coli* K-12 which was sensitive to the detergent (Fig. 5, data of wild-type not shown). The mechanism of resistance of APEC could be both LPS and plasmid related. In fact, the detection of plasmid-associated resistance in *E. coli* K-12 background but not in the wild-type background strains, could be related to the presence of other factors, including the LPS in these strains that has masked the effect of plasmids on this phenomenon; this statement is supported by the resistance of the rough mutant which is usually hypersensitive to bile [57]. The mechanism of resistance encoded by the plasmid pChi7122-1 could be associated with proteins such as OmpT that was previously associated with bile resistance in *Vibrio cholerae* [58] and ABC transport proteins that are known to play a role in the protection of cells from toxic compounds [59]. Since such factors are not located on pChi7122-2, other factors predicted to be encoded by this plasmid, such as TA modules could be involved in bile tolerance of bacteria; as TA systems are now known to play an important role in bacterial stress physiology [60], [61], [62]. To our knowledge, this is the first time that plasmids have been shown to be associated with the bile resistance of *E. coli*.

Acid resistance is important for bacterial survival in acidic stomach or in foods with low pH [63]. Our results have shown that plasmids do not have any effect on the growth of the wild-type derived strains when grown in acidic medium for a short period (12 hours), as the strains with and without plasmids grew similarly (data not shown). However, at longer incubation times (18 hours), strains behaved differently (Fig. 5B). Similar to the study by Lim *et al*. [64] on the plasmid pO157 in *E. coli* O157, we have shown that in the absence of its three plasmids, the APEC strain survived better in acidic conditions than in their presence when incubated for 18 h. Moreover, our study showed that although the plasmid pChi7122-1, either alone or in combination with pChi7122-2 or pChi7122-3, decreased the acid tolerance of bacteria, the presence of pChi7122-3 had the opposite effect (Fig. 5B). Since pChi7122-1 and pO157 play a major role in the virulence of APEC [12] and *E. coli* O157 [64] respectively, these findings could indicate that the presence of plasmids exert a cost to bacterial fitness when exposed for a long period (>18 hours) to acidic conditions, whereas bacteria containing other plasmids such as pChi7122-3 in χ7122 would have better survivability in these conditions. Elucidation of the mechanism of acid tolerance associated with pChi7122-3 is needed to fully understanding the persistence of *E. coli* in acidic conditions.

Our study also confirmed the importance of the full expression of O78-antigen LPS for the acid tolerance of *E. coli* [65], and demonstrated that the nature of LPS had a minor effect on this stress response (Fig. 5B).

#### Large plasmids increase biofilm formation at host temperatures

Bacterial biofilm formation is a major concern in both medical and industrial systems. Biofilm formation is associated with many medically-important pathogenic bacteria, as an estimated 65-80% of all human infections are thought to be biofilm-related [66]. However, elucidating the mechanisms of biofilm formation necessary for establishing strategies for their prevention and treatments is becoming a matter of urgency.

ExPEC cells are found in biofilm-like communities in both gastrointestinal [67] and urinary tracts [68] indicating the importance of biofilms in the persistence of these bacteria. ExPEC bacteria have to adapt to extreme temperature changes. In this study, our strategy using three large plasmids, either individually or in combination in both an APEC wild-type and an *E. coli* K-12 background, and different O-LPS at different temperatures, has revealed new insights into biofilm formation of ExPEC. Altogether, our data distinguished four groups of factor-driven biofilms, including plasmidless-, plasmid-, O-LPS-, and rough LPS-mediated biofilms in *E. coli* which differ in their expression conditions.

**Figure 6:**
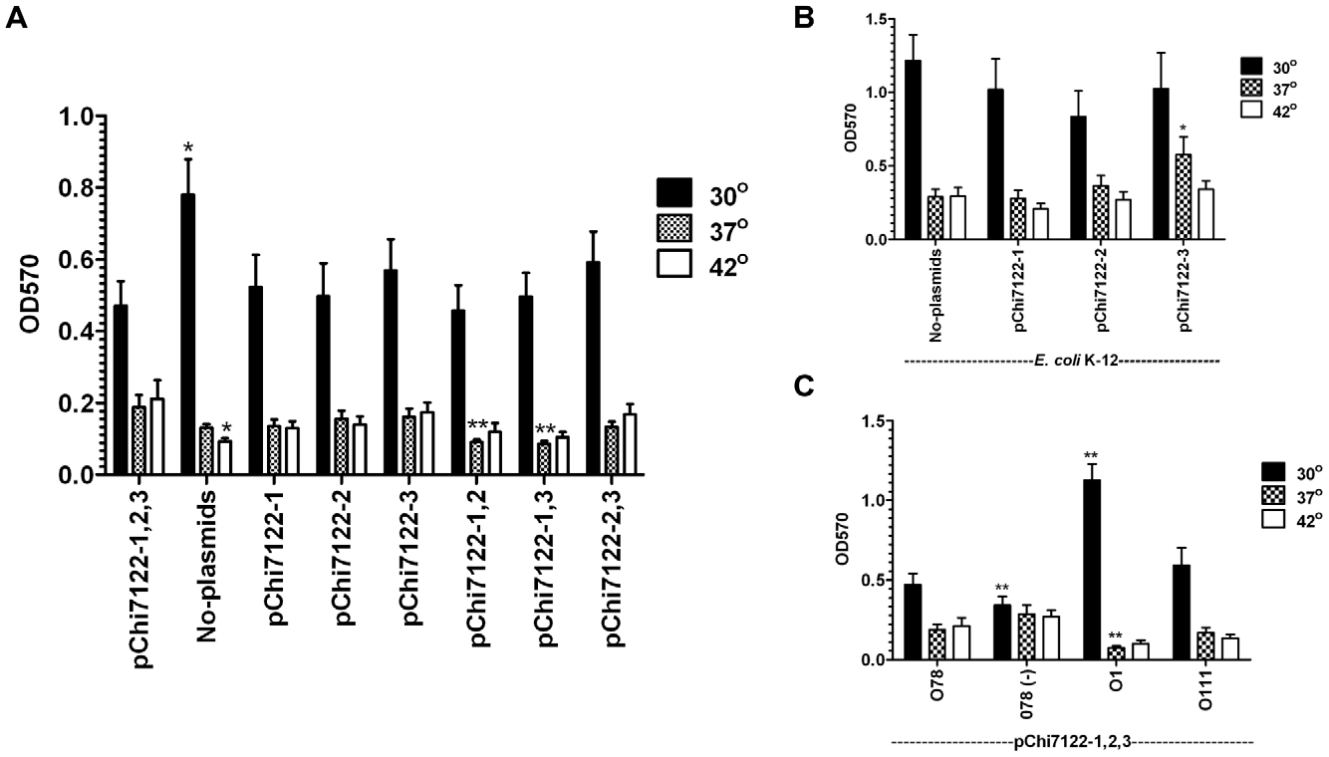
Effect of plasmids, O-LPS, and temperature on biofilm formation. Biofilm formation of different strains were compared at different temperatures: χ7122 and its plasmid derivatives strains: No-plasmids (χ7368), pChi7122-1 (χ7394), pChi7122-2 (χ7392), pChi7122-3 (χ7367), pChi7122-1,2 (χ7561), pChi7122-1-3 (χ7562), and pChi7122-2,3 (χ7274) (**A**), *E. coli* K-12 (χ6092) and its derivatives: pChi7122-1 (χ7346), pChi7122-2 (χ7347), and pChi7122-3 (χ7348) (**B**), and χ7122 and its LPS derivative strains : rough mutant O78(-) (χ7145), smooth strains with either O1-LPS (O1) (χ7193) or O111-LPS (O111) (χ7167) (**C**) at either 30°C, 37°C, or 42°C.

In general, the different strains tested formed more biofilms at 30°C than at 37°C or 42°C (Fig. 6A). Compared to the wild-type, the plasmidless strain produced significantly more biofilm at 30°C (*P*<0.05) (Fig. 6A). In the same conditions, the presence of the three plasmids, either individually or in combinations in the strains, reduced the level of biofilm formation to the level of the wild-type strain (Fig. 6A). In contrary, at host temperatures (37° and 42°) (Fig. 6A), the plasmidless strain produced less biofilm than the wild-type strain, with the data being statistically significant (*P*<0.05) at 42°C (Fig. 6A).

The biofilm formed by the plasmid-cured strain, highly produced at 30°C (Fig. 6A), is probably promoted by no-plasmidic factors preferentially expressed at 30°C and at early stage of biofilm formation; among them curli required for development of biofilm and adhesion [69]. Expression of biofilm in the environment (30°C) would be beneficial for plasmidless strains; in these conditions, biofilm will allow these bacteria to be in close proximity with other bacterial species and acquire transmissible genetic elements.

It has been shown that conjugative plasmids promote bacterial biofilm formation by generating F-pili mating pairs, which is important for early biofilm formation [70], [71], [72]. In this report, we have shown that plasmid-driven biofilms are very complex and this complexity is related to the nature of the plasmids, their combinations, host strain backgrounds, and the temperature to which the strains are exposed. The presence of the three plasmids pChi7122-1, pChi7122-2, and pChi7122-3 in the wild-type strain (Fig. 6A) and pChi7122-3 in the *E. coli* K-12 strain (Fig. 6B), had increased biofilm formation at host temperature conditions, with data being significant at 42°C (*P*<0.05) (Fig. 6B). The fact that pChi7122-3-driven enhancement of bacterial biofilm was higher than those of pChi7122-2 and pChi7122-1 in both wild-type and the *E. coli* K-12 backgrounds could be related to not only the *tra* genes expression [70], [71], [72] but also to the type IV fimbriae encoded by pChi7122-3, which was previously associated with the biofilm formation in enteroaggregative *E. coli* [73]. Plasmid-driven biofilms could be essential in the virulence process by giving bacteria a survival advantage in different niches of the host, which could result in disease.

A controversy exists regarding the role of LPS in bacterial biofilm formation [74], [75]. In this study, we have shown that the three plasmids pChi7122-1, pChi7122-2, and pChi7122-3 in wild-type derivative strains with different O-LPS backgrounds behaved differently in their biofilm formation (Fig. 6C). In absence of O78-LPS, the rough strain produced significantly (*P*<0.0001) less biofilm than its smooth wild-type strain at 30°C. Even though substitution of O78-LPS with O111-LPS had little effect on biofilm formation, the substitution of O78-LPS with O1-LPS has in contrary greatly enhanced biofilm formation in these bacteria at 30°C. Since the O1-LPS-driven enhancement of bacteria biofilm occurs at 30°C condition and is repressed at host temperatures (37°C/42°C), this indicates that its role could be more important in the persistence of bacteria in the environment, and that the temperature of 30°C in early O1-LPS-associated biofilm formation is necessary. The fact that O1-LPS-driven biofilm is not highly formed at 37°C and 42°C (Fig. 6C), could be related to a change in the LPS-O1 bilayer structure at higher temperature [76], [77], [78] leading to the disturbance of the early biofilm formed. To our knowledge, this is the first report on the effect of the nature of LPS on biofilm formation.

### Conclusion

A novel putative sugar utilization pathway operon that is not present in other *E. coli* strains but found in *Salmonella* serovars, an ABC iron transport system and a type IV fimbriae *pil* operon were located on pChi7122-2 and pChi7122-3 respectively. Multiple plasmid-encoded mechanisms, including toxin-antitoxin modules and the novel sugar pathway could be important in the fitness and persistence of APEC χ7122.

Large plasmids were involved in bile resistance (pChi7122-1 and pChi7122-2) when present in *E. coli* K-12 background and acid tolerance (pChi7122-3) in the wild-type background. Four different factor-driven biofilms, including plasmidless-, plasmid-, rough-LPS-, and O-LPS-mediated were demonstrated. These multiple factor-driven biofilms expressed at different temperatures could have distinct functions. Some of them could be important in the acquisition of genetic material and persistence of bacteria in the environment; others could be involved in virulence. The genotypic and phenotypic analysis of plasmid-derivative strains of an ExPEC model strain χ7122 (O78∶K80∶H9) revealed new insights into the mechanisms of fitness of ExPEC and their diversity.

## Materials and Methods

### Bacterial strains and growth conditions

Most of the bacterial strains used in this study, listed in Table 1, are derived from the highly virulent APEC strain χ7122 (O78∶K80∶H9) [79] and were fully described in our previous studies [10], [12], [80].

To evaluate the effect of the host strain background on plasmid-associated phenotypes, we used three derivatives of χ7122 with different LPS profiles containing the three plasmids pChi7122-1, pChi7122-2, and pChi7122-3; a rough mutant strain (O78-) of APEC χ7122, χ7145; and two derivatives of χ7145, χ7167 and χ7193, which respectively express O111 and O1 antigens rather than the native O78 antigen. We also used strains derived from an *E. coli* K-12, χ6092, containing either pChi7122-1, χ7346; pChi7122-2, χ7347; or pChi7122-3, χ7348 (Table 1, [10], [12], [80]).

Antibiotic susceptibility testing of strains was performed and interpreted via disk diffusion method, as recommended by the Clinical and Laboratory Standards Institute (CLSI) [81], [82].

A collection of one hundred human strains isolated from the main clinical extra-intestinal sources (50 UTI and 50 non-UTI) [19], eighty APEC strains, and forty-five enteric *E. coli* strains (from our collection) were used to study the distribution of pChi7122-2 and pChi7122-3-associated genes among different groups of pathogenic *E. coli* by PCR.

Unless otherwise stated, bacteria were routinely grown in Luria Bertani (LB) broth or on MacConkey agar supplemented with 1% lactose at 37°C. Strains were stored as stock cultures at −80°C in peptone-glycerol medium. All wild-type derivative strains grew similarly in LB and reached the OD_600_ ∼1.0 when incubated overnight (O/N) at 37°C standing, except the strain χ7561 (pChi7122-1, pChi7122-2) which had a slightly lower growth and its OD_600_ was around 0.8 when grown in the same conditions.

### Plasmid sequencing and annotation

The DNA sequences of pChi7122-2, pChi7122-3 and pChi7122-4 plasmids were derived from contig sequences of the whole genomic DNA of APEC χ7122. The sequences were manipulated to the standard of an ‘Improved High-Quality Draft’ [83]. The program Artemis [84] was used to identify the plasmids and collate data. For each of the three plasmids all the sequence gaps were closed by directed polymerase chain reaction (PCR) and the products sequenced with big dye terminator chemistry on ABI3730 capillary sequencers. All the plasmids were circularized and contiguated using this method.

The DNA sequences were annotated to identify coding sequences and repeat sequences in Artemis and the vector NTI suite of programs was used to confirm the previous analysis. To identify plasmids with similar sequences, pChi7122-2 (FR851303) and pChi7122-3 (FR851304) were compared by BLASTn at NCBI. Plasmid sequences of pEG356 (FN594520.1) from *Shigella sonnei*, pHK01 (HM355591.1) from a urinary *E. coli* isolate, pAA (FN554767.1) from EAEC, and pKF3-70 (FJ494913.1) from *K. pneumoniae*, and R721 (AP002527.1) a trimethoprim and streptomycin resistant plasmid from an *E. coli* were downloaded. Plasmid sequences were aligned and then visualized using ACT and Mauve v2.3.1 [85]. Schematic plasmid drawings were constructed using DNAplotter [86].

Amino acid sequence and protein structural-based alignments were performed using the CLC Free Workbench software tool (v. 6.1 CLC bio A/S, Aarhus, Denmark) and the web-based interface for ESPript v.2.2 (http://espript.ibcp.fr/ESPript/cgi-bin/ESPript.cgi) [87], respectively. The 3-D structure of the proteins pChiA, pChiT, pChiO, pChiD, and pChiR were predicted using position specific iterative (PSI)- BLAST alignment and HHpred [38].

### Prevalence of pChi7122-2 (*eitA*) and pChi7122-3 (*pilS* and pilV) genes among avian and human pathogenic *E. coli*

One hundred human *E. coli* strains isolated from the main clinical extra-intestinal sources (50 UTI and 50 non-UTI) [19], eighty APEC strains, and forty-five enteric *E. coli* strains (from our collection) were screened by PCR [19] for the presence of the *eitA*, *pilS*, and *pilV* genes using the primers *eitA* F: 5′-AACTGCGGCTATCAGGAGAC-3′ and *eitA* R : 5′-CAGGTCATATCCCACAGCTT-3′; *pilS* F: 5′-CTTCTCTTTCTGCACACCGT-3′ and *pilS* R: 5′-TGTGATTGTAACGGAGCC-3′; *pilV* F: 5′-TCTATACAGGCGAGTATTTA-3′ and *pilV* R: 5′-AATTCATACCAGAATACTCA-3′. The primers were designed from the sequence of pChi7122-2 (*eitA*) and pChi7122-3 (*pilS* and *pilV*).

### Growth comparison assays

The growth rates of strains were compared in iron-sequestered medium using LB containing 100 µM of α,α′-dipyridyl alone or supplemented with either Heme (Sigma, 600 µg ml^−1^), hemoglobin (Sigma, 60 µg ml^−1^), or FeSO_4_ (Sigma, 20 mM: control). The growth rates were also tested in minimal medium MM9 [12], MM9 with thiamin (1 µg ml^−1^) and 0.1% casamino acid, and supplemented with glucose, lactose, arabinose, mannose, galactose, glucuronic acid, or glucoronic acid (0.2%) as source of carbon at 37°C shaking (180 rpm). The OD_600_ was recorded every hour over 24-hour period.

### Interaction of strains with 3-D INT-407 human epithelial cells

3-D cultures of human INT-407 cells (ATCC CCL6) were used as model intestinal epithelium and were prepared as previously described [54]. Approximately 10^6^ CFU of PBS-washed bacteria, grown rotating to an OD_600_ 1.0 in LB, were added to each well (multiplicity of infection [MOI], 10). For bacterial association assays, the 24-well plates were incubated at 37°C in 5% CO_2_ for 1 hour, and rinsed three times with PBS. PBS-0.1% (w/v) deoxycholic acid sodium salt was added to each well, and samples were diluted and spread on MacConkey medium plates for enumeration by viable colony counting. For invasion assays, extracellular bacteria were killed following the initial 1-h incubation period by an additional 1-h incubation in medium containing gentamicin (100 µg/ml; Sigma-Aldrich). Cells were then washed 3× with PBS and lysed. Bacterial titers in the lysates were determined by serial dilutions and plating on MacConkey agar. The results were expressed as the Log_10_CFU/ml.

Antibodies specific for O78-LPS (Denken Seiken) and the human tight junction protein ZO-1 (Invitrogen) were used for confocal laser scanning microscopy (CLSM) imaging. Antibodies were of porcine and mouse origins, respectively, and were used at a dilution of 1∶500 (anti-O78-LPS) and 1∶100 (anti-ZO-1). Goat anti-porcine and anti-mouse secondary antibodies labeled with Alexa Fluor 555 (Invitrogen) were used to detect the bound primary antibodies anti-O78-LPS and anti-ZO-1 respectively and were diluted 1∶500 in blocking solution (8% bovine serum albumin, 0.05% Triton-X100 in DPBS). Cell nuclei and the F-actin cytoskeleton were visualized with 4′, 6-diamidino-2-phenylindole hydrochloride (DAPI) and phalloidin (Invitrogen), respectively. The fixation and staining of 3-D aggregates was performed as described previously [88]. Optical sections of the 3-D aggregates were obtained using a Zeiss LSM 510 Duo laser scanning microscope equipped with detectors and filter sets for monitoring emissions of the selected fluorophores. Images were acquired using a Plan-Neofluar 40×/1.3 oil DIC objective and were analyzed with the Zeiss LSM software package. Axiovision 4.8 software from Carl Zeiss was used to further process collected images.

### Sensitivity of strains to deoxycholate (DOC) and acid shock tolerance

To determine the ability of bacteria to survive at sub-lethal bile concentration, different strains were grown rotating to an OD_600_ 1.0 in LB medium. Five-microliters of serial ten-fold dilutions of each strain were spotted on both LB agar and LB agar containing 1% (w/v) DOC plates and incubated overnight at 37°C.

For acid shock assays, bacterial cells were grown at 37°C in LB broth, pH 7, O/N standing. Cultures adjusted to the same OD_600_ of 1.0 were diluted 1∶1,000 in LB, pH 2.5, and incubated at 37°C with gentle shaking (50 rpm). Samples were cultured by direct plating on LB agar after 8 h and 18 h to determine the percent survival following acid stress. As controls, bacteria were also grown in LB, pH 7 in the same conditions to determine if the growth of bacteria was affected.

### Biofilm formation assay

Biofilm formation assays were performed in 96-well polystyrene microtiter plates (Becton Dickinson, Franklin Lakes, NJ) [89]. In brief, strains were grown to stationary phase in LB at 37°C and then diluted 1∶100 in LB supplemented with 0.1% (w/v) L-glucose. Aliquots of 200 µL for each dilution were dispensed per well into a microtiter plate (four wells/strain). Each strain was tested in quadruplicate, wells containing sterile medium were used as negative controls. Plates were sealed with parafilm and cultured standing either at 30°C, 37°C or 42°C for 5 days to mimic the environmental and body temperature of humans and chickens, respectively. The media of the plates were then decanted, and the plates were washed twice with sterile PBS. Microplates were then stained with 200 µL of 1% (w/v) Crystal Violet for 30 min, followed by washing twice with PBS to remove unbound dye. After drying, dye-containing adherent cells were resolubilized with 200 µL of 30% (v/v) acetic acid solution. The absorbance was measured at 570 nm in an ELISA reader (SpectraMax M2, Molecular Devices). All tests were carried out at least three times, and the results were averaged.

### Statistical analysis

Data were analyzed by one-way analysis of variance (ANOVA), followed by Bonferroni's multiple-comparison test (GraphPad Prism software, version 5.07). Differences between average values were also tested for significance by performing an unpaired, two-sided Student *t* test. The levels of significance (*P* values) are reported and values ≤0.05 were taken to be significant.

## Supporting Information

Figure S1 **Plasmids genomes comparison.** Mauve pairwise nucleotide comparison of the complete pChi7122-2 DNA sequence to that of pEG356 (FN594520.1), pHK01 (HM355591.1), pAA (FN554767.1), and pKF3-70 (FJ494913.1) (**A**) and pChi7122-3 DNA sequence to that of R721 (AP002527.1). The colored boxes represent homologous segments completely free of genomic rearrangements. These boxes are connected by lines between genomes. Blocks below the center line indicate regions with inverse orientation. Regions outside blocks lack homology between genomes. White regions indicate the sequence specific to a genome.
(TIF)

Figure S2 **Comparison of growth rates of bacteria in iron-restricted media.** *E. coli* K-12 (χ6092) and its derivatives: pChi7122-1 (χ7346), pChi7122-2 (χ7347), and pChi7122-3 (χ7348) were grown in LB medium containing 2,2′-dipyridyl (- iron) or supplemented with either FeSO_4_ (control), Heme, or Hemoglobin at 37°C for 24 h.
(TIF)

Figure S3 **Multiple amino acid sequence alignment.** pChiD, pChiO, pChiT, and pChiA of pChi7122-2 were aligned with their homologous proteins from other bacteria. Arrows indicate β sheets; spirals α helixes and TT loops.
(TIF)

Figure S4 **Comparison of growth rates of bacteria in the presence of different carbon sources.** The wild-type strain χ7122 and its derivatives: No-plasmids (χ7368), pChi7122-1 (χ7394), pChi7122-2 (χ7392), pChi7122-3 (χ7367) were tested for growth in either strict MM9 (**A**) or MM9 containing thiamin and casamino acid (**B**) without sugar, or with different sugars (glucose, lactose, arabinose, mannose, galactose, glucoronic acid, or glucoronic acid).
(TIF)

Table S1 **Summary of information about the coding sequences of pChi7122-2.** In this table, we present details of all coding sequences found in pChi7122-2.
(DOC)

Table S2 **Summary of information about the coding sequences of pChi7122-3.** In this table, we present details of all coding sequences found in pChi7122-3.
(DOC)

Table S3 **Putative functions of pChi7122-2-encoded sugar pathways genes.** In this table, we present the putative functions of *pChiA*, *pChiD*, *pChiT*, *pChiO*, and *pChiR* genes of the sugar pathway encoded by pChi7122-2.
(DOC)
