# Supplementary figures and images for: New Insights into the Bacterial Fitness-Associated Mechanisms Revealed by the Characterization of Large Plasmids of an Avian Pathogenic E. coli
Source: PLoS One. 2012 Jan 4;7(1):e29481. doi: 10.1371/journal.pone.0029481 (PMC3251573; doi:10.1371/journal.pone.0029481)

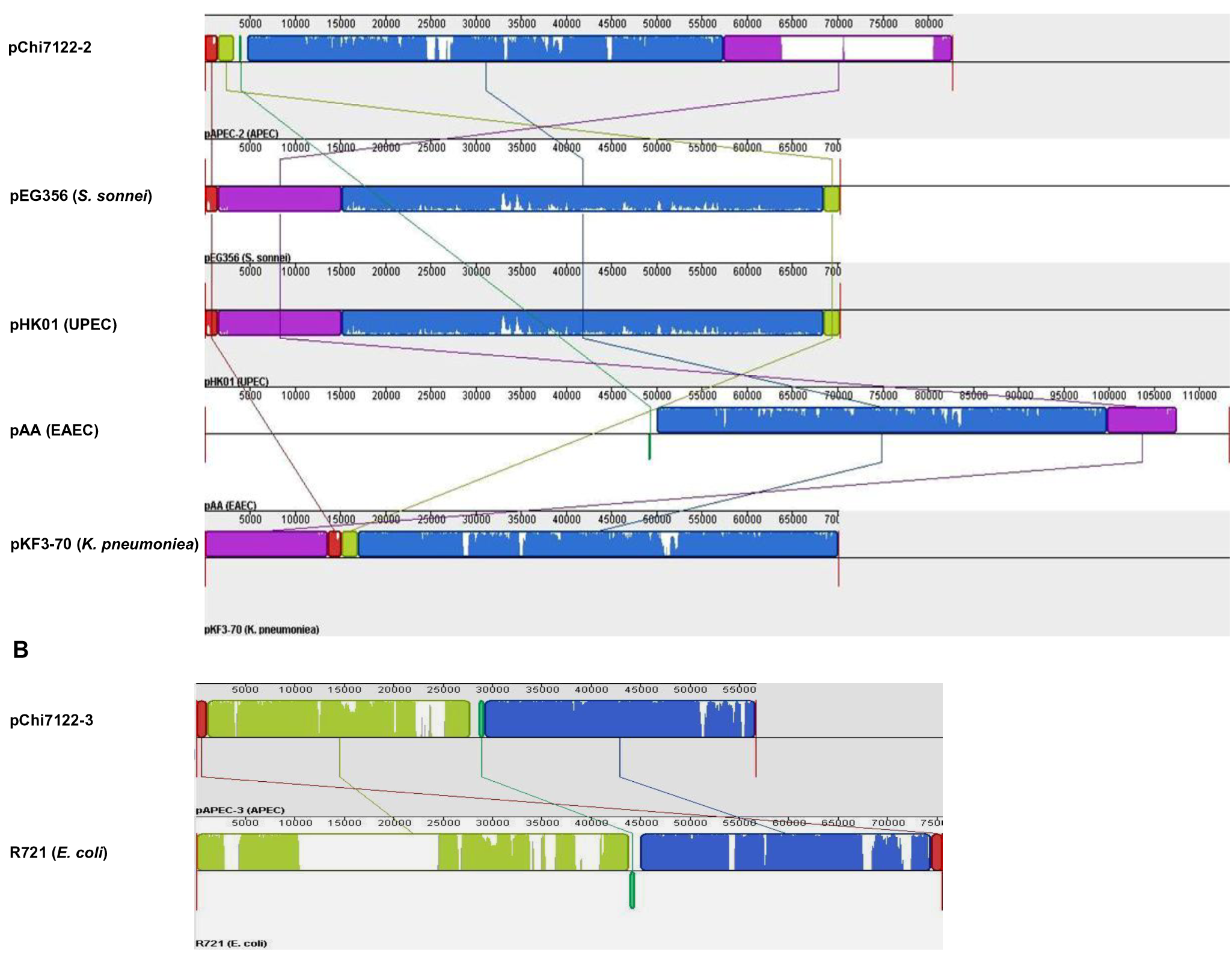

Supplement: Figure S1 — Plasmids genomes comparison. Mauve pairwise nucleotide comparison of the complete pChi7122-2 DNA sequence to that of pEG356 (FN594520.1), pHK01 (HM355591.1), pAA (FN554767.1), and pKF3-70 (FJ494913.1) (A) and pChi7122-3 DNA sequence to that of R721 (AP002527.1). The colored boxes represent homologous segments completely free of genomic rearrangements. These boxes are connected by lines between genomes. Blocks below the center line indicate regions with inverse orientation. Regions outside blocks lack homology between genomes. White regions indicate the sequence specific to a genome. (TIF) [file pone.0029481.s001.tif]

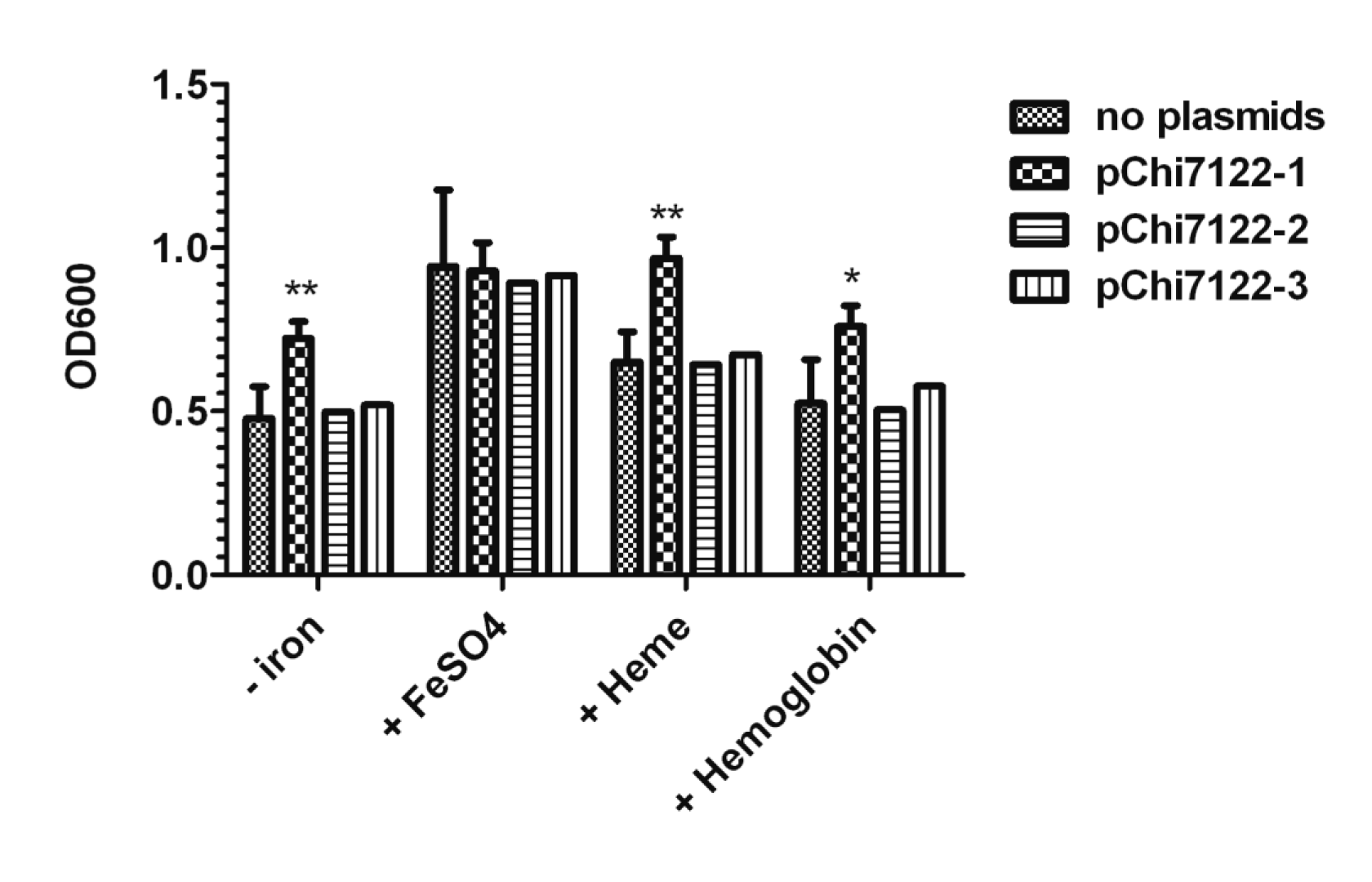

Supplement: Figure S2 — Comparison of growth rates of bacteria in iron-restricted media. E. coli K-12 (χ6092) and its derivatives: pChi7122-1 (χ7346), pChi7122-2 (χ7347), and pChi7122-3 (χ7348) were grown in LB medium containing 2,2′-dipyridyl (- iron) or supplemented with either FeSO4 (control), Heme, or Hemoglobin at 37°C for 24 h. (TIF) [file pone.0029481.s002.tif]

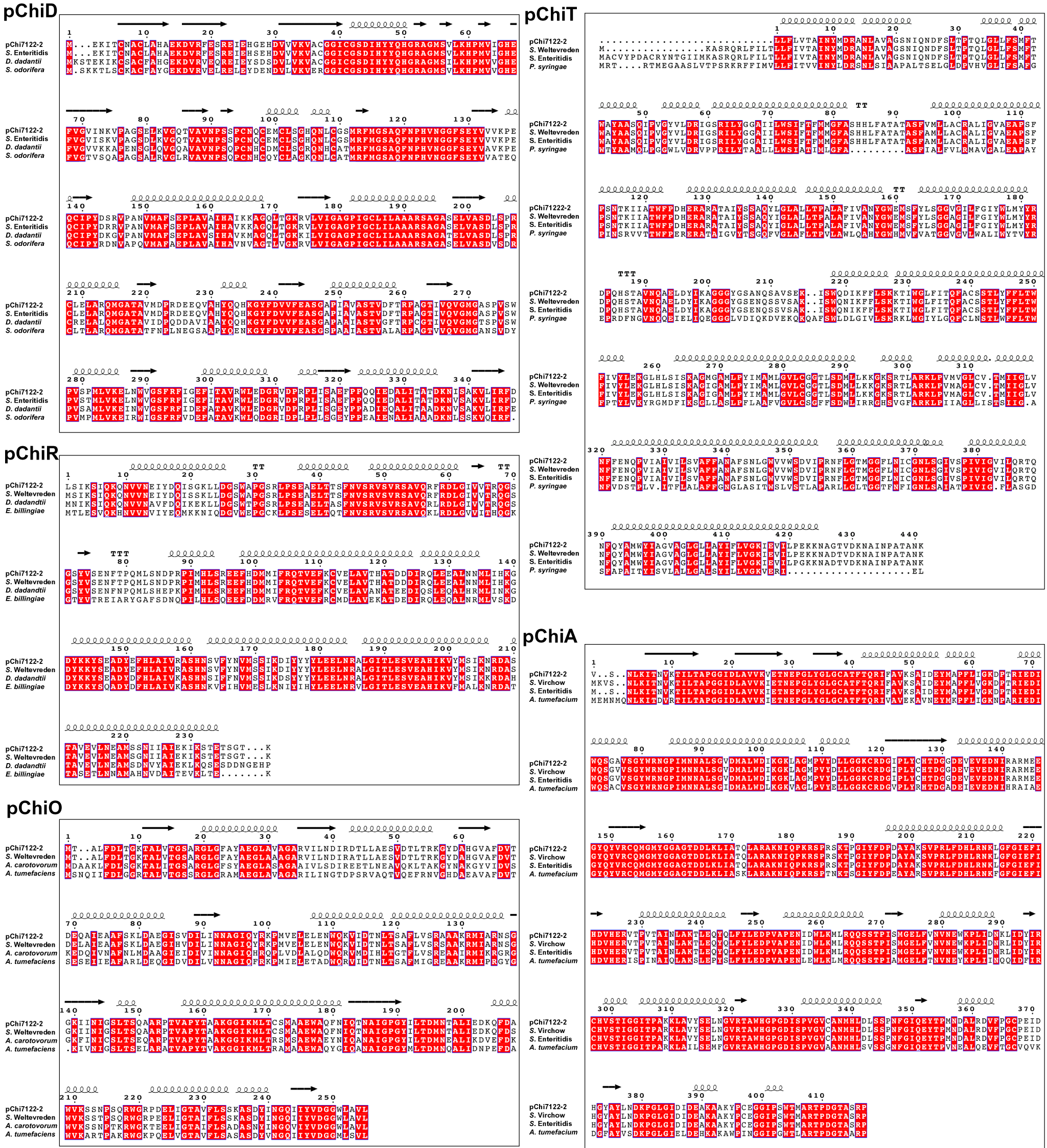

Supplement: Figure S3 — Multiple amino acid sequence alignment. pChiD, pChiO, pChiT, and pChiA of pChi7122-2 were aligned with their homologous proteins from other bacteria. Arrows indicate β sheets; spirals α helixes and TT loops. (TIF) [file pone.0029481.s003.tif]

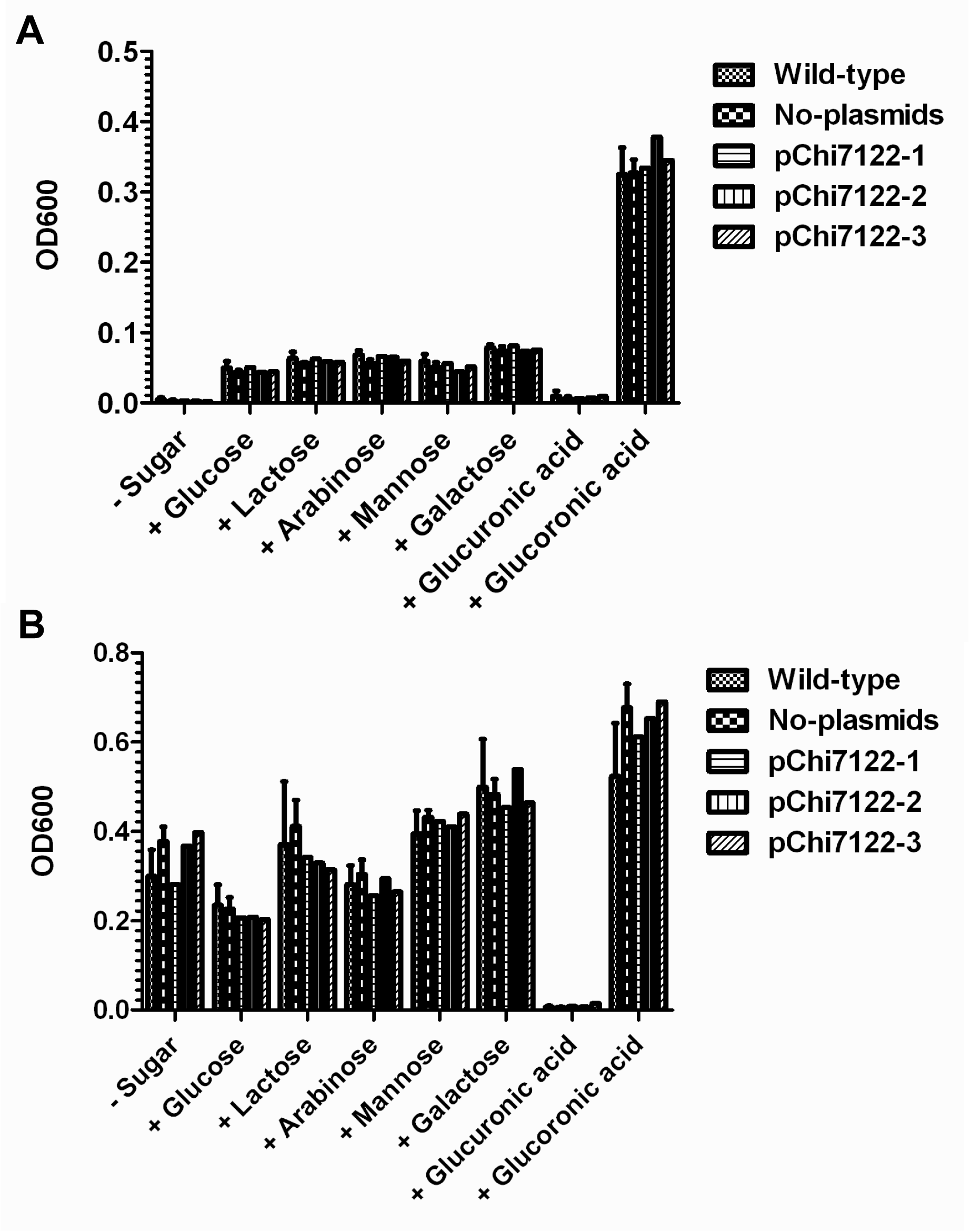

Supplement: Figure S4 — Comparison of growth rates of bacteria in the presence of different carbon sources. The wild-type strain χ7122 and its derivatives: No-plasmids (χ7368), pChi7122-1 (χ7394), pChi7122-2 (χ7392), pChi7122-3 (χ7367) were tested for growth in either strict MM9 (A) or MM9 containing thiamin and casamino acid (B) without sugar, or with different sugars (glucose, lactose, arabinose, mannose, galactose, glucoronic acid, or glucoronic acid). (TIF) [file pone.0029481.s004.tif]
