# Supplementary material for: New Insights into the Bacterial Fitness-Associated Mechanisms Revealed by the Characterization of Large Plasmids of an Avian Pathogenic E. coli
Source: PLoS One. 2012 Jan 4;7(1):e29481. doi: 10.1371/journal.pone.0029481 (PMC3251573; doi:10.1371/journal.pone.0029481)
Supplement: Table S1 — Summary of information about the coding sequences of pChi7122-2. In this table, we present details of all coding sequences found in pChi7122-2. (DOC) [file pone.0029481.s005.doc]

**Table S1**. Summary of information about the coding sequences of pChi7122-2

| **GI Number** | **Position (bp)** | **Orientation** | **Size (AA)** | **Gene symbol** | **Gene function** | **Expect Score** | **% identity** | **GeneBank reference** |
| --- | --- | --- | --- | --- | --- | --- | --- | --- |
| MM2_001 | 1-687 | - | 228 | *repA1* | IncFIIA replication protein repA1 | 1e-128 | 98(ETEC) | BAI49252 |
| MM2_002 | 791-970 | + | 59 | *repA3* | Replication protein repA3 | 1e-21 | 87(APEC) | YP_001481220 |
| MM2_003 | 987-1235 | - | 82 | *copB* | Copy number control protein | 3e-39 | 100(ETEC) | YP_003232549 |
| MM2_004 | 1475-2065 | - | 196 |  | yihA | 2e-107 | 100 (R100) | NP_052987 |
| MM2_005 | 2104-2313 | - | 69 |  | Hemolysin expression-modulating protein | 2e-32 | 100(R100) | YP_053130 |
| MM2_006 | 2359-2880 | - | 173 |  | Staphylococcal nuclease homologue family protein | 4e-93 | 95(*E. coli*) | YP_001919353 |
| MM2_007 | 3018-3251 | - | 77 |  | CHP | 6e-25 | 73(*S.* *sonnei*) | YP_313453 |
| MM2_008 | 3332-3448 | - | 38 |  | HP |  |  |  |
| MM2_009 | 3720-3842 | - | 40 |  | HP |  |  |  |
| MM2_010 | 3920-4597 | - | 225 |  | HP | 6e-129 | 100(EPEC) | YP_003232545 |
| MM2_011 | 4884-5096 | - | 70 |  | yigA | 2e-30 | 98(*E. coli*) | ZP_04533050 |
| MM2_012 | 5227-5787 | - | 186 | *finO* | Conjugal transfer fertility inhibition protein FinO | 1e-102 | 100(*Shigella*) | YP_311530 |
| MM2_013 | 5842-6588 | - | 248 | *traX* | F pilin acetylase | 7e-139 | 99(ETEC) | YP_003294035 |
| MM2_014 | 6608-11878 | - | 1756 | *traI* | DNA helicase I | 0.0 | 98(*E. coli*) | AAQ98619 |
| MM2_015 | 11878-14067 | - | 729 | *traD* | Conjugal transfer protein TraD | 0.0 | 97(*E. coli*) | YP_003237809 |
| MM2_016 | 14320-15075 | - | 252 | *traT* | Conjugal transfer surface exclusion protein TraT | 3e-142 | 100(*E. coli*) | CBG27823 |
| MM2_017 | 15083-15586 | - | 167 | *traS* | Surface exclusion inner membrane protein TraS | 9e-93 | 98(*E. coli*) | YP_002401089 |
| MM2_018 | 15601-18423 | - | 940 | *traG* | Conjugal transfer mating pair stabilization protein TraG | 0.0 | 98(*E. coli*) | ZP_03069972 |
| MM2_019 | 18420-19793 | - | 457 | *traH* | Conjugal transfer pilus assembly protein TraH | 0.0 | 98 (*E. coli*) | YP_788085 |
| MM2_020 | 19780-20172 | - | 130 | *trbF* | Conjugal transfer protein TrbF | 9e-70 | 99(ETEC) |  |
| MM2_021 | 20153-20434 | - | 93 | *trbJ* | Conjugal transfer protein TrbJ | 4e-41 | 93(UPEC F11) | ZP_03035140 |
| MM2_022 | 20424-20969 | - | 181 | *trbB* | F pilus assembly periplasmic protein TrbB | 2e-100 | 98(*E. coli*) | YP_538728 |
| MM2_023 | 20956-21240 | - | 94 | *traQ* | Conjugal transfer pilin chaperone TraQ | 2e-45 | 98(*E. coli*) | NP_052971 |
| MM2_024 | 21367-21705 | - | 112 | *trbA* | Conjugal transfer protein TrbA | 2e-56 | 99(EAEC) | YP_788080 |
| MM2_025 | 21721-22464 | - | 247 | *traF* | Conjugal pilus assembly protein TraF | 8e-143 | 99(ETEC) | YP_002456210 |
| MM2_026 | 22457-22714 | - | 85 | *trbE* | Conjugal transfer protein TrbE | 2e-40 | 97(pO86A1) | YP_788078 |
| MM2_027 | 22741-24591 | - | 616 | *traN* | Conjugal transfer mating pair stabilization protein TraN | 0.0 | 97(*E. coli*) | NP_957618 |
| MM2_028 | 24588-25010 | - | 140 |  | HNH endonuclease | 3e-77 | 100(EAEC) | ZP_03069975 |
| MM2_029 | 25035-25406 | - | 123 |  | HP | 1e-65 | 99(EAEC) | ZP_03070076 |
| MM2_030 | 25403-26041 | - | 212 | *trbC* | Type F conjugative transfer system pilin assembly protein TrbC | 2e-117 | 96(*E. coli*) | ZP_03030161 |
| MM2_031 | 26234-26473 | + | 79 |  | HP | 3e-37 | 100(ETEC) | ZP_03030166 |
| MM2_032 | 26470-26646 | - | 58 |  | HP | 1e-26 | 100(ETEC) | ZP_03030152 |
| MM2_033 | 26602-26781 | + | 59 |  | HP |  |  |  |
| MM2_034 | 26910-27050 | - | 46 |  | HP |  |  |  |
| MM2_035 | 27113-27421 | - | 102 |  | CHP | 6e-41 | 97(EAEC) | ZP_03071987 |
| MM2_036 | 27448-28440 | - | 330 | *traU* | Conjugal transfer pilus assembly protein TraU | 0.0 | 100(K. *pneumoniae*) | YP_003329213 |
| MM2_037 | 28437-29069 | - | 210 | *traW* | Conjugal transfer pilus assembly protein TraW | 2e-118 | 99(EAEC) | ZP_03072257 |
| MM2_038 | 29066-29371 | - | 101 | *trbI* | Conjugal transfer protein TrbI | 2e-51 | 100(EPEC O26) | YP_003232521 |
| MM2_039 | 29449-32076 | - | 875 | *traC* | F pilus assembly protein TraC | 0.0 | 99(*E. coli*) | YP_003108312 |
| MM2_040 | 32236-32457 | - | 73 | *traR* | Conjugal transfer protein TraR | 3e-35 | 98(*E. coli*) | YP_003108311 |
| MM2_041 | 32592-33107 | - | 171 | *traV* | Conjugal transfer protein TraV | 2e-94 | 100(*E. coli*) | YP_003108310 |
| MM2_042 | 33104-33355 | - | 83 | *trbG* | Conjugal transfer protein TrbG | 2e-40 | 100(*E. coli*) | YP_190137 |
| MM2_043 | 33348-33668 | - | 106 | *trbD* | Conjugal transfer protein TrbD | 2e-55 | 100(*E. coli*) | YP_003108308 |
| MM2_044 | 33655-34239 | - | 194 | *traP* | Conjugal transfer protein TraP | 5e-111 | 100(*E. coli*) | YP_003108307 |
| MM2_045 | 34229-35656 | - | 475 | *traB* | Conjugal transfer pilus assembly protein TraB | 0.0 | 99(*E. coli*) | YP_003108306 |
| MM2_046 | 35656-35787 | - | 43 | *traK-like* |  | 1e-16 | 97(EAEC) | ZP_03069984 |
| MM2_047 | 35784-36293 | - | 169 | *traK* | Conjugal transfer protein TraK | 1e-91 | 100(K. *pneumoniae*) | ADD63611 |
| MM2_048 | 36383-36508 | - | 41 |  | HP |  |  |  |
| MM2_049 | 36549-36914 | - | 121 | *traE* | Conjugal transfer pilus assembly protein TraE | 7e-57 | 99(*E. coli*) | YP_788064 |
| MM2_050 | 36936-37247 | - | 103 | *traL* | Conjugal transfer pilus assembly protein TraL | 2e-52 | 98(*E. coli*) | NP_061454 |
| MM2_051 | 37262-37501 | - | 79 | *traA* | Conjugal transfer pilin subunit TraA | 3e-36 | 98(EAEC) | CBG27849 |
| MM2_052 | 37660-37842 | - | 60 | *traY* | Plasmid nicking protein | 4e-26 | 100(UPEC) | YP_538704 |
| MM2_053 | 37981-38667 | - | 228 | *traJ* | Functional relaxosome complex initiator | 1e-130 | 99(UPEC) | YP_538703 |
| MM2_054 | 38861-39244 | - | 127 | *traM* | Conjugal transfer protein TraM | 9e-68 | 100(*E. coli*) | YP_003108299 |
| MM2_055 | 39530-40177 | + | 215 |  | Transglycosylase SLT domain-containing protein | 2e-119 | 95(*E. coli*) | YP_003108298 |
| MM2_056 | 40474-41295 | - | 273 |  | CHP | 8e-159 | 99( EAEC) | YP_001451573 |
| MM2_057 | 41405-41701 | - | 98 |  | HP | 1e-50 | 100(R100) | NP_052941 |
| MM2_058 | 41724-41864 | - | 46 |  | CHP | 1e-16 | 95(*K*.pneumoniae) | YP_003329236 |
| MM2_059 | 41966-42178 | - | 70 |  | ydaA | 7e-31 | 100(EAEC) | CBG27867 |
| MM2_060 | 42471-42617 | + | 48 |  | HP | 5e-13 | 86(*E. coli*) | YP_003108285 |
| MM2_061 | 42690-42932 | - | 80 |  | HP | 4e-40 | 100(K.pneumoniae) | YP_003329239 |
| MM2_062 | 42991-43302 | - | 103 |  | CHP | 2e-54 | 100(*K*.pneumoniae) | YP_003329240 |
| MM2_063 | 43268-43462 | - | 64 |  | CHP | 3e-15 | 97(O157) | ZP_03085374 |
| MM2_064 | 43466-43609 | + | 47 |  | CHP | 1e-19 | 100(*K*.pneumoniae) | YP_003329241 |
| MM2_065 | 43785-44051 | - | 88 |  | HP | 3e-18 | 95(EPEC) | ZP_03062951 |
| MM2_066 | 44159-44320 | - | 53 | *hok* | Post-segregation killing protein | 1e-22 | 100(*E. coli*) | YP_424869 |
| MM2_067 | 44496-45014 | - | 172 |  | Similar to Upf89.0 (entrobacteria phage P7, Stx2-conerting phage) | 7e-98 | 99(*E. coli*) | YP_001919403 |
| MM2_068 | 45014-45733 | - | 239 | *psiA* | Plasmid SOS inhibition protein A | 1e-132 | 97(plasmid R100, F) | NP_061443 |
| MM2_069 | 45730-46167 | - | 145 | *psiB* | Plasmid SOS inhibition protein B | 7e-79 | 98(APEC) | YP_001481172 |
| MM2_070 | 46219-48177 | - | 652 | *parB* | ParB-like partition protein | 0.0 | 96(EAEC) | ZP_03072152 |
| MM2_071 | 48241-48474 | - | 77 |  | CHP | 2e-38 | 100(K.pneumoniae) | YP_003329248 |
| MM2_072 | 48530-49057 | - | 175 | *ssb* | Single-stranded DNA-binding protein | 4e-97 | 100(*E. coli*) | YP_001919398 |
| MM2_073 | 49083-49223 | - | 46 |  | CHP | 5e-17 | 97(K.pneumoniae) | YP_003329236 |
| MM2_074 | 49325-49537 | - | 70 |  | ydaA | 3e-30 | 97(K.*pneumoniae*) | ADD63621 |
| MM2_075 | 49948-50511 | - | 187 |  | unkwon | 2e-104 | 99(*E. coli*) | CBG27870 |
| MM2_076 | 50558-51919 | - | 453 |  | ydbA | 0.0 | 99(EAEC) | ZP_03070012 |
| MM2_077 | 51971-52201 | - | 76 |  | ydfB | 5e-35 | 100(UPEC) | YP_538685 |
| MM2_078 | 52294-52434 | - | 46 |  | HP | 5e-18 | 97(UPEC) | ZP_03035122 |
| MM2_079 | 52460-52717 | + | 85 |  | CHP | 1e-43 | 100(*E. coli*) | BAI58021 |
| MM2_080 | 52748-53029 | + | 93 |  | HP | 2e-41 | 94(K.pneumoniae) | ADD63637 |
| MM2_081 | 53238-53429 | - | 63 |  | 98 pct identical to gp:AF106329_[orf63 of plasmid F] | 2e-29 | 100(UPEC) | YP_538680 |
| MM2_082 | 53426-53848 | - | 140 |  | ycjA | 2e-72 | 98(EPEC) | ZP_03063040 |
| MM2_083 | 53895-54320 | - | 141 |  | Antirestriction protein klcA | 5e-79 | 100(*E. coli*) | YP_788038 |
| MM2_084 | 54569-54736 | - | 55 |  | CHP | 4e-23 | 98(K.pneumoniae) | YP_003329261 |
| MM2_085 | 54736-55506 | - | 256 |  | ychA ta | 6e-145 | 99(EAEC) | CBG27880 |
| MM2_086 | 55551-55985 | - | 144 |  | CHP | 5e-76 | 97(ETEC) | YP_002456147 |
| MM2_087 | 55999-56220 | - | 73 |  | unkown | 3e-34 | 100(*S*. Typhimurium) | YP_194815 |
| MM2_088 | 56221-56904 | - | 227 |  | DNA methylase family protein | 7e-132 | 99(EPEC) | ZP_03051147 |
| MM2_089 | 57012-57137 | + | 41 |  | HP | 6e-14 | 97 (*K*.pneumoniae) | YP_003329266 |
| MM2_090 | 57421-58383 | + | 320 | *stbA* | Stable plasmid inheritance protein A | 0.0 | 99(EAEC) | CBG27885 |
| MM2_091 | 58386-58736 | + | 116 | *stbB* | Stable plasmid inheritance protein B | 4e-61 | 100(*K*.pneumoniae) | YP_003329173 |
| MM2_092 | 58908-59054 | + | 48 |  | HP | 9e-20 | 100(*K*.pneumoniae) | YP_003329174 |
| MM2_093 | 59587-60585 | + | 332 | *eitA* | Putative iron transport system, periplasmic binding protein | 0.0 | 99(APEC) | YP_001481257 |
| MM2_094 | 60585-61622 | + | 345 | *eitB* | Iron compound ABC superfamily ATP binding cassette transporter | 0.0 | 100(*K*.pneumoniae) | ZP_06017231 |
| MM2_095 | 61622-62383 | + | 253 | *eitC* | Iron(III) dicitrate transport ATP-binding | 1e-145 | 100(*K*.pneumoniae) | YP_002919938 |
| MM2_096 | 62395-63627 | + | 410 | *eitD* | ABC iron transporter system; permease protein | 0.0 | 100 (S. *sonnei*) | YP_003377717 |
| MM2_097 | 63705-63821 | - | 38 |  | Colicin E2 immunity protein | 9e-11 | 91(pAPEC-O2-ColV) | YP_444145 |
| MM2_098 | 63861-64565 | - | 234 |  | IS1216 transposase | 2e-136 | 100 (S. Choleraesuis) | YP_209330 |
| MM2_099 | 64566-64925 | + | 119 |  | intIdelta1 protein | 2e-38 | 95(Corynebacterium glutamicum | NP_478098 |
| MM2_100 | 64982-65581 | - | 199 |  | CHP (putative transposase) | 1e-107 | 100(*E. coli*) | YP_001688196 |
| MM2_101 | 66641-67480 | - | 279 | *Sul1* | Dihydropteroate synthase | 1e-156 | 99(*E. coli*) | CAC87991 |
| MM2_102 | 66013-66513 | - | 166 |  | GCN5-related N-acetyl transferase | 2e-92 | 100(bacterium S5) | ZP_06404987 |
| MM2_103 | 67985-68992 | - | 335 | *aadA* | Streptomycin 3”-adenylytransferase (SP-R) | 0.0 | 100(ETEC) | ZP_03028124 |
| MM2_104 | 69772-70494 | - | 240 |  | IS1216 transposase | 8e-140 | 99(S. Choleraesuis) | YP_209330 |
| MM2_105 | 70738-73725 | - | 995 | *tnpA* | Transposase for transposon Tn2501 | 0.0 | 97(S. Saintpaul) | ZP_02347600 |
| MM2_106 | 73893-74534 | + | 213 | *tnpR* | Resolvase N terminal domain protein | 4e-116 | 95(*K*. pneumoniae) | YP_002235570 |
| MM2_107 | 74628-75881 | - | 417 | *pChiA* | Starvation-sensing protein rspA | 0.0 | 98(S.Enteritidis) | YP_002243537 |
| MM2_108 | 76219-76986 | + | 255 | *pChiO* | Gluconate 5-dehydrogenase | 6e-140 | 100(S. Weltevreden) | ZP_02831409 |
| MM2_109 | 77106-78431 | + | 441 | *pChiT* | Major facilitator superfamily protein | 0.0 | 97(S.Weltevreden) | ZP_02831410 |
| MM2_110 | 78448-79491 | + | 347 | *pChiD* | L-idonate 5-dehydrogenase | 0.0 | 97(S. Enteritidis) | YP_002243534 |
| MM2_111 | 79582-80301 | + | 239 | *pChiR* | Putative GntR domain protein | 8e-137 | 98(S.Weltevreden) | ZP_02831412 |
| MM2_112 | 80872-81171 | - | 99 |  | Putative plasmid stabilization system | 3e-47 | 96(*E. coli*) | YP_001919362 |
| MM2_113 | 81161-81424 | - | 87 |  | HP | 6e-33 | 96(*E. coli*) | YP_001919361 |
| MM2_114 | 81531-81791 | - | 56 |  | HP | 4e-19 | 85(ETEC) | BAI49251 |
| MM2_115 | 81883-82131 | - | 82 | *repA4* | Replication protein RepA4 | 4e-35 | 90(APEC) | YP_443946 |
| MM2_116 | 82494-82679 | - | 60 | *repA1* | Replication initiation protein RepA1 (IncF11) | 1e-25 | 85(ETEC) | YP_003294026 |

-, Reverse; +, forward , AA, amino acid; GI, GenInfo Identifier; AA, amino acid; Hp, hypothetical protein; CHP, conserved hypothetical protein.
