# Supplementary material for: New Insights into the Bacterial Fitness-Associated Mechanisms Revealed by the Characterization of Large Plasmids of an Avian Pathogenic E. coli
Source: PLoS One. 2012 Jan 4;7(1):e29481. doi: 10.1371/journal.pone.0029481 (PMC3251573; doi:10.1371/journal.pone.0029481)
Supplement: Table S2 — Summary of information about the coding sequences of pChi7122-3. In this table, we present details of all coding sequences found in pChi7122-3. (DOC) [file pone.0029481.s006.doc]

**Table S2.** Summary of information about the coding sequences of pChi7122-3

| **GI Number** | **Position (bp)** | **orientation** | **Size (AA)** | **Gene Symbol** | **Gene Function** | **% Identity** | **Expect** | **GeneBank reference** |
| --- | --- | --- | --- | --- | --- | --- | --- | --- |
| MM3_001 | 76-582 | - | 168 |  | HP | 100 (R721) | 1e-94 | NP_065383 |
| MM3_002 | 777-1073 | - | 98 |  | HP | 100 (R721) | 4e-51 | NP_065384 |
| MM3_003 | 1226-1585 | - | 119 |  | HP |  |  |  |
| MM3_004 | 1631-1687 | + | 18 | *repR* | Replication initiation leader sequence | 100 (*E. coli*) | 2e-08 | NP_065291 |
| MM3_005 | 1668-2696 | + | 341 | *repA* | Replication initiation protein | 99 (O157) | 0.0 | ZP_05940622 |
| MM3_006 | 2794-3117 | - | 107 |  | HP |  |  |  |
| MM3_007 | 3260-3421 | + | 53 | *repA4* | Replicaton protein A4 | 84 (R100) | 7e-17 | NP_052991 |
| MM3_008 | 3527-3640 | **+** | 114 |  | HP |  |  |  |
| MM3_009 | 3678-3932 | - | 84 |  | HP |  |  |  |
| MM3_010 | 4132-4452 | + | 106 | *mok* | Modulator of post-segregation killing protein | 77(R721) | 5e-33 | YP_002401058 |
| MM3_011 | 4606-5202 | + | 198 | *finO* | FinO superfamily | 100 (R721) | 6e-110 | NP_065298 |
| MM3_012 | 5220-5504 | - | 94 |  | Stability/partitioning determinant | 100(R721) | 1e-47 | NP_065299 |
| MM3_013 | 5578-6243 | - | 221 | *parA* | Plasmid partition protein A | 100(*E. coli*) | 5e-125 | NP_065300 |
| MM3_014 | 6352-6690 | - | 112 |  | HP |  |  |  |
| MM3_015 | 6736-7170 | + | 144 |  | CHP | 98(R721) | 6e-57 | NP_065301 |
| MM3_016 | 7279-7425 | - | 48 |  | HP |  |  |  |
| MM3_017 | 7450-7665 | + | 71 |  | HP |  |  |  |
| MM3_018 | 7752-8075 | + | 107 |  | HP | 99(R721) | 6e-55 | NP_065302 |
| MM3_019 | 8116-8325 | + | 69 |  | Similar to pir:T14933 [Y1017 of plasmid pMT1] | 98(R721) | 3e-30 | NP_065303 |
| MM3_020 | 8422-8646 | + | 74 |  | HP | 83(O157) | 2e-28 | YP_002268559 |
| MM3_021 | 8698-8958 | + | 86 |  | HP | 97(O157) | 4e-41 | ZP_03086083 |
| MM3_022 | 9111-9422 | - | 103 |  | HP |  |  |  |
| MM3_023 | 9445-9597 | - | 50 |  | HP |  |  |  |
| MM3_024 | 9597-9755 | - | 52 |  | HP |  |  |  |
| MM3_025 | 9750-10028 | + | 92 |  | [CcgD-like of plasmid pKM101](http://manatee.igs.umaryland.edu/tigr-scripts/chado_prok_manatee/shared/btab_display.cgi?db=pAPEC3&file=/local/dmz/aengine/search_data/aengine/output_repository/ber/1632146331_pre_overlap_analysis/i1/g1/pAPEC3.polypeptide.1632166308.1.wu-blastp.ber.nr.btab&orf=pAPEC3.transcript.1632166309.1&accession=GB:BAB12600.1) | 98(R721) | 5e-46 | NP_065307 |
| MM3_026 | 10113-10232 | + | 39 |  | HP |  |  |  |
| MM3_027 | 10337-10714 | + | 125 | *hicB* | HicB Family | 100(R721) | 1e-43 | NP_065321 |
| MM3_028 | 10821-11264 | - | 147 |  | Unknown protein | 100(R721) | 1e-79 | NP_065322 |
| MM3_029 | 11484-11627 | - | 47 |  | HP |  |  |  |
| MM3_030 | 11768-12205 | + | 145 |  | CHP [Uncharacterized protein family (UPF0150) family protein] | 100(R721)/O157 | 1e-80 | NP_065324 |
| MM3_031 | 12336-12662 | - | 108 |  | Similar to EaA protein of bacteriophage P22 | 96(O157) | 3e-52 | ZP_05940602 |
| MM3_032 | 12682-13281 | - | 199 |  | HP | 100(R721) | 1e-108 | NP_065327 |
| MM3_033 | 13328-14029 | - | 233 |  | CHP | 100(R721) | 3e-131 | NP_065328 |
| MM3_034 | 14035-14514 | - | 159 |  | HP | 100(R721) | 1e-86 | NP_065329 |
| MM3_035 | 14844-15185 | + | 113 | *nikA* | Bacterial mobilization protein (MobC) family | 100 (O157) | 5e-59 | ZP_03085905 |
| MM3_036 | 15182-18949 | + | 1255 | *nikB* | Relaxase | 79 (O157) | 0.0 | ZP_05940593 |
| MM3_037 | 16158-16457 | + | 89 | *nikC* | Relaxosome accessory protein | 100 (R721) | 7e-51 | NP_065332 |
| MM3_038 | 18992-19432 | - | 146 |  | Putative membrane protein | 96(O157) | 7e-73 | ZP_05940592 |
| MM3_039 | 19481-19636 | - | 51 |  | HP |  |  |  |
| MM3_040 | 19828-19983 | - | 51 |  | Transcriptional regulator | 86(O157) | 3e-07 | ZP_03085810 |
| MM3_041 | 20052-22217 | - | 721 | *topB* | DNA topoisomerase III | 95(R721) | 0.0 | NP_065340 |
| MM3_042 | 22229-22816 | - | 195 |  | HP | 87(O157) | 2e-92 | ZP_02783794 |
| MM3_043 | 23180-23584 | - | 134 |  | HP |  |  |  |
| MM3_044 | 23600-23752 | - | 50 | *parB* | Uncharacterized endonuclease (ORFA) domain protein | 96(*S.* dysenteriae) | 2e-21 | ZP_03067536 |
| MM3_045 | 23905-24345 | + | 146 |  | putative transposase | 97 (*E. coli*) | 2e-80 | YP_002389672 |
| MM3_046 | 24342-25112 | + | 256 |  | Transposase, IS609 OrfB family | 97(ETEC) | 2e-145 | YP_003221916 |
| MM3_047 | 25135-25350 | - | 71 |  | nuclease domain protein | 97(O157) | 1e-29 | ZP_05940586 |
| MM3_048 | 25397-26050 | - | 217 |  | CHP | 99(O157) | 3e-124 | ZP_03086240 |
| MM3_049 | 26062-27186 | - | 394 |  | Shufflon-specific DNA recombinase | 98(O157) | 0.0 | ZP_05940584 |
| MM3_050 | 27225-27602 | + | 125 |  | Shufflon protein A. domain protein | 100(R721) | 1e-55 | A45252 |
| MM3_051 | 27780-28094 | + | 104 |  | Shufflon protein B’. domain protein | 50(R64) | 2e-17 | P09748 |
| MM3_052 | 28603-28941 | + | 112 |  | PilV variable region D' | 100(R721) | 1e-47 | F45252 |
| MM3_053 | 28951-30237 | - | 428 | *pilV* | Minor pilin subunit PilV | 99(R721) | 0.0 | NP_065346 |
| MM3_054 | 30250-30885 | - | 211 | *pilU* | Type IV leader peptidase family protein | 100(R721) | 6e-115 | NP_065347 |
| MM3_055 | 30889-31371 | - | 160 | *pilT* | Lytic transglycosylase PilT | 99(R721) | 2e-88 | NP_065348 |
| MM3_056 | 31438-31995 | - | 185 | *pilS* | Type IV prepropilin PilS | 97(R721) | 6e-91 | NP_065349 |
| MM3_057 | 32040-33149 | - | 369 | *pilR* | Bacterial type II secretion system protein F domain protein. | 99(R721) | 0.0 | NP_065350 |
| MM3_058 | 33140-34678 | - | 512 | *pilQ* | Type II/IV secretion system family protein (pilus biogenesis ATPase PilQ) | 99(O157) | 0.0 | ZP_05940654 |
| MM3_059 | 34703-35197 | - | 164 | *pilP* | Type IV pilus biogenesis periplasmic protein PilP | 99(R721) | 1e-88 | NP_065352 |
| MM3_060 | 35181-36503 | - | 440 | *pilO* | Type IV pilus biogenesis outer membrane protein PilO | 99(R721) | 0.0 | NP_065353 |
| MM3_061 | 36542-38185 | - | 547 | *pilN* | Type IV pilus outer membrane secretin lipoprotein PilN | 100 (R721) | 0.0 | NP_065354 |
| MM3_062 | 38236-40077 | - | 613 | *traK* | Conjugal transfer protein TraK | 98 (O157) | 0.0 | ZP_05940649 |
| MM3_063 | 40209-41264 | - | 351 | *traJ* | Conjugal transfer protein TraJ | 100 (R721) | 0.0 | NP_065357 |
| MM3_064 | 41283-42422 | - | 379 | *traI* | Conjugal transfer protein TraI | 99 (R721) | 0.0 | NP_065358 |
| MM3_065 | 42412-43182 | - | 256 | *traH* | Conjugal transfer protein TraH | 99(R721) | 2e-149 | NP_065359 |
| MM3_066 | 43179-43913 | - | 244 | *traG* | Conjugal transfer protein TraG | 99 (R721) | 4e-138 | NP_065360 |
| MM3_067 | 44075-46432 | - | 785 | *traE* | Conjugal transfer protein TraE | 98(R721) | 0.0 | NP_065362 |
| MM3_068 | 46438-46758 | - | 106 | *traD* | TraD protein | 100(O157) | 4e-53 | ZP_03085719 |
| MM3_069 | 46829-47119 | - | 96 | *traC* | Conjugal transfer prepropilin TraC | 100(R721) | 8e-47 | NP_065364 |
| MM3_070 | 47119-47703 | - | 194 | *traB* | Conjugal transfer protein TraB | 99 (R721) | 2e-108 | NP_065365 |
| MM3_071 | 47724-48122 | - | 132 |  | HP | 100 (R721) | 2e-69 | NP_065366 |
| MM3_072 | 48241-48678 | - | 145 | *pilM* | Type IV pilus biogenesis protein PilM | 100 (R721) | 5e-80 | NP_065367 |
| MM3_073 | 48684-49919 | - | 411 | *pilL* | Type IV pilus biogenesis outer membrane protein precursor PilL | 99 (R721) | 0.0 | NP_065368 |
| MM3_074 | 49922-50209 | - | 95 |  | KikA-like of plasmid pKM101, a protein that appears to increase lethality when the plasmid is conjugationally transferred to Klebsiella cells; killing in Klebsiella phenotype | 98(R721) | 2e-47 | NP_065369 |
| MM3_075 | 50381-51016 | - | 211 |  | TrcD-like of plasmid ColIb-P9] | 99(O157) | 1e-118 | ZP_05940635 |
| MM3_076 | 51089-51376 | - | 95 |  | unknown | 100(*E. coli*) | 5e-47 | ZP_07244618.1 |
| MM3_077 | 51389-51643 | - | 84 |  | unknown | 100(*E. coli*) | 1e-40 | ZP_07244619 |
| MM3_078 | 51645-52286 | - | 213 | *trbJ* | Conjugal transfer protein TrbJ | 94(R721) | 1e-108 | NP_065373 |
| MM3_079 | 52292-53287 | - | 331 | *traA* | Conjugal transfer protein TraA | 90(R721) | 7e-168 | NP_065374 |
| MM3_080 | 53291-53548 | - | 85 |  | HP | 100(R721) | 5e-42 | NP_065375 |
| MM3_081 | 53545-53847 | - | 100 |  | HP | 95(R721) | 2e-39 | NP_065376 |
| MM3_082 | 53880-54085 | - | 71 |  | HP | 100(O157) | 4e-28 | ZP_03086055 |
| MM3_083 | 54118-54564 | - | 148 |  | HP | 100(O157) | 1e-79 | ZP_03086054 |
| MM3_084 | 54575-54745 | - | 56 |  | Putative membrane protein | 100(O157) | 2e-22 | ZP_03086053 |
| MM3_085 | 54749-55192 | - | 147 |  | NfeD-like family protein | 96(O157) | 5e-76 | ZP_05940627 |
| MM3_086 | 55566-56543 | - | 325 |  | Putative protease stomatin/prohibitin homologs | 100(O157) | 0.0 | ZP_03086599 |

-, Reverse; +, forward, AA, amino acid; GI, GenInfo Identifier; AA, amino acid; Hp, hypothetical protein; CHP, conserved hypothetical protein.
