# Supplementary material for: New Insights into the Bacterial Fitness-Associated Mechanisms Revealed by the Characterization of Large Plasmids of an Avian Pathogenic E. coli
Source: PLoS One. 2012 Jan 4;7(1):e29481. doi: 10.1371/journal.pone.0029481 (PMC3251573; doi:10.1371/journal.pone.0029481)
Supplement: Table S3 — Putative functions of pChi7122-2-encoded sugar pathways genes. In this table, we present the putative functions of pChiA, pChiD, pChiT, pChiO, and pChiR genes of the sugar pathway encoded by pChi7122-2. (DOC) [file pone.0029481.s007.doc]

**Table S3. Putative functions of pChi7122-2-encoded sugar pathways genes**

| **Gene** | **Protein** | **Similar polypeptide** | **Identity** | **Species** | **Nucleotide accession number** |
| --- | --- | --- | --- | --- | --- |
| *pChiA* | pChiA | D-altronate/D-mannonate dehydratase | 98% | *Salmonella* Enteritidis | YP_002243537.1 |
|  |  | Mandelate racemase/muconate lactonizing enzyme | 98% | *Salmonella* Virchow | ZP_03216462.1 |
|  |  | Galactonate dehydratase | 78% | *Escherichia fergusonii* | EGC96352.1 |
| *pChiD* | pChiD | L-idonate 5-dehydrogenase | 97% | *Salmonella* Enteritidis | YP_002243534 |
|  |  | Alcohol dehydrogenase GroES domain protein | 81% | *Dickeya dadantii* | YP_002986573 |
|  |  | L-idonate 5-dehydrogenase | 71% | *Serratia odorifera* | ZP_06190913.1 |
| *pChiT* | pChiT | Probable glucarate transporter | 97% | *Salmonella* Weltevreden | ZP_02831410.1| |
|  |  | Exonate sugar transport protein | 97% | *Salmonella* Enteritidis | YP_002243535.1 |
|  |  | D-galactonate transporter | 43% | *Pseudomonas syringae* | YP_235071.1 |
| *pChiO* | pChiO | Gluconate 5-dehydrogenase | 96% | *Salmonella* Weltevreden | ZP_02831409.1 |
|  |  | Putative hexonate dehydrogenase | 69% | *Pectobacterium carotovorum* | ZP_03830676.1 |
|  |  | gluconate dehydrogenase | 67% | *Agrobacterium tumefaciens* | NP_354414 |
| *pChiR* | pChiR | putative GntR domain protein | 98% | *Salmonella* Weltevreden | ZP_02831412.1 |
|  |  | regulatory protein GntR | 83% | *Dickeya dadantii* | YP_002986572.1 |
|  |  | Transcriptional regulator, GntR family | 62% | *Erwinia billingiae* | YP_003741600.1 |
